# Deficiency in Th2 Cytokine Responses Exacerbate Orthopoxvirus Infection

**DOI:** 10.1371/journal.pone.0118685

**Published:** 2015-03-09

**Authors:** Isaac G. Sakala, Geeta Chaudhri, Preethi Eldi, R. Mark Buller, Gunasegaran Karupiah

**Affiliations:** 1 Infection and Immunity Group, Department of Immunology, John Curtin School of Medical Research, Australian National University, Canberra, ACT, Australia; 2 Department of Molecular Microbiology and Immunology, Saint Louis University Health Sciences Center, St Louis, MO, United States of America; Federal University of São Paulo, BRAZIL

## Abstract

Ectromelia virus (ECTV) causes mousepox in mice, a disease very similar to smallpox in humans. ECTV and variola virus (VARV), the agent of smallpox, are closely related orthopoxviruses. Mousepox is an excellent small animal model to study the genetic and immunologic basis for resistance and susceptibility of humans to smallpox. Resistance to mousepox is dependent on a strong polarized type 1 immune response, associated with robust natural killer (NK) cell, cytotoxic T lymphocyte (CTL) and gamma interferon (IFN-γ) responses. In contrast, ECTV-susceptible mice generate a type 2 response, associated with weak NK cell, CTL and IFN-γ responses but robust IL-4 responses. Nonetheless, susceptible strains infected with mutant ECTV lacking virus-encoded IFN-γ binding protein (vIFN-γbp) (ECTV-IFN-γbp^Δ^) control virus replication through generation of type 1 response. Since the IL-4/IL-13/STAT-6 signaling pathways polarize type 2/T helper 2 (Th2) responses with a corresponding suppression of IFN-γ production, we investigated whether the combined absence of vIFN-γbp, and one or more host genes involved in Th2 response development, influence generation of protective immunity. Most mutant mouse strains infected with wild-type (WT) virus succumbed to disease more rapidly than WT animals. Conversely, the disease outcome was significantly improved in WT mice infected with ECTV-IFN-γbp^Δ^ but absence of IL-4/IL-13/STAT-6 signaling pathways did not provide any added advantage. Deficiency in IL-13 or STAT-6 resulted in defective CTL responses, higher mortality rates and accelerated deaths. Deficiencies in IL-4/IL-13/STAT-6 signaling pathways significantly reduced the numbers of IFN-γ producing CD4 and CD8 T cells, indicating an absence of a switch to a Th1-like response. Factors contributing to susceptibility or resistance to mousepox are far more complex than a balance between Th1 and Th2 responses.

## Introduction

Smallpox was one of the biggest human scourges when it was rife, with mortality rates of 30–40%. VARV is an orthopoxvirus (OPV) that causes smallpox, a disease that has killed more humans than all other infectious diseases combined [1–4]. As the disease was eradicated more than 30 years ago, animal models of OPV infections have been used to establish strong causal relationships and map the requirements for protection against infection in a way that is not possible in humans. ECTV is a natural pathogen of mice that causes a generalized infection known as mousepox, an excellent small animal model to study the pathogenesis of smallpox [4–7]. Both VARV and ECTV cause acute infections in their respective hosts, which either succumb to infection or clear the virus with complete recovery.

Resistance to mousepox is dependent on rapid generation of a strong polarized type 1 immune response [8]. ECTV-resistant C57BL/6 mice generate robust NK cell, CTL, and IFN-γ responses whereas in ECTV-susceptible BALB/c mice these responses are sub-optimal but high levels of IL-4 is produced. Loss of IFN-γ function in resistant mice [9,10] or over-expression of IL-4 by recombinant ECTV encoding IL-4, which abrogates IFN-γ production [11,12] results in ineffective NK cell and CTL responses and fulminant disease. The existence of virus-encoded host response modifiers that specifically target type 1 cytokines further underscores the importance of these factors in immunity to poxvirus infections [13–16]. Indeed, BALB/c mice infected with a deletion mutant virus lacking vIFN-γbp (ECTV-IFN-γbp^Δ^) are able to contain virus replication and overcome the infection [17]. Absence of vIFN-γbp increases IFN-γ production and as a consequence, augments cell-mediated immunity and virus control.

The cytokine milieu and cell subset-selective transcription factors play important roles in the differentiation of uncommitted naive CD4 T cells into Th1 and Th2 subsets following antigenic stimulation [18,19]. IFN-γ and IL-4 play dominant roles in the development of Th1 and Th2 subsets, respectively. IL-4 mediates its activity through the IL-4 receptor (R), which consists of a heterodimeric complex between IL-4Rα and the common γ chain [20,21]. IL-4Rα also associates with the low affinity IL-13Rα1 to form a high affinity receptor for both IL-13 and IL-4. A second IL-13 receptor, IL-13Rα2, which exists in a monomeric form, can bind to IL-13 with high affinity and modulate IL-13 responses [22,23]. Binding of IL-4 to IL-4R activates the transcription factor STAT-6, which in turn controls expression of GATA3, the master transcription regulator required for Th2 differentiation [24]. IL-13 is also capable of activating STAT-6 and shares many of the biological properties of IL-4.

A previous report pointed to a role for STAT-6 signaling and IL-4 production in contributing to susceptibility of BALB/c mice to ECTV infection [25]. STAT-6 deficient mice infected with ECTV-WT were better protected through generation of more effective NK cell and IFN-γ responses that allowed the otherwise susceptible animals to overcome infection. Curiously, IL-4 deficiency in BALB/c mice has no impact on disease outcome [8], implying that other factors like IL-13 may be sufficient to drive Th2-like responses in the absence of IL-4.

Since the generation of protective immunity and the outcome of a viral infection are influenced by both viral and host genes, we hypothesized that infection of gene knockout (GKO) BALB/c mice lacking one or more factors associated with Th2 response development with ECTV-WT or ECTV-IFN-γbp^Δ^ would further augment the Th1 response and influence disease outcome. Contrary to our prediction, the disease was exacerbated in most groups of BALB/c mice lacking one or more components of Th2 signaling pathways compared to WT mice infected with WT virus. Conversely, the disease outcome was significantly improved in WT mice infected with the mutant virus, but absence of the IL-4/IL-13/STAT-6 signaling pathways did not provide any added advantage to the host. In fact, there were higher rates of mortality and accelerated deaths amongst mice deficient in IL-13 or STAT-6 compared with WT animals. Deficiency in IL-13 or STAT-6 dramatically reduced the antiviral CTL response. Furthermore, deficiencies in IL-4/IL-13/STAT-6 signaling pathways significantly reduced the numbers of IFN-γ producing CD4 and CD8 T cells, indicating an absence of a switch to a Th1-like response. Thus, although recovery from ECTV infection is strongly influenced, in part, by a balance between the host’s ability to produce IFN-γ and the virus’ ability to dampen its effects [17], our data indicate that factors contributing to disease susceptibility are far more complex than a balance between Th1 and Th2 responses.

## Materials and Methods

### Ethics Statement

This study was performed in strict accordance with the recommendations in the Australian code of practice for the care and use of animals for scientific purposes and the Australian National Health and Medical Research Council Guidelines and Policies on Animal Ethics and approved by the Animal Ethics and Experimentation Committee of the Australian National University (Protocol numbers J.IG. 62/08, J.IG. 75.09 and A2012/041).

### Mice

Gene knockout (GKO) mice lacking IL-4 [26], IL-13 [27], IL-4Rα [28], STAT-6 [29] or both IL-13 and IL-4Rα were either generated on a BALB/c background or backcrossed to the BALB/cJ strain for at least 10 generations or more. Female BALB/c WT and GKO mice designated IL-4^−/−^, IL-13^−/−^, STAT-6^−/−^, IL-4Rα^−/−^, IL-13^−/−^/IL-4Rα^−/−^ and were bred under specific-pathogen-free conditions at the Australian Phenomics Facility, Australian National University and used at 6–8 weeks of age. The IL-4^−/−^ mice can respond to IL-13 whereas IL-13^−/−^ mice have intact IL-4 responses. IL-4Rα^−/−^ mice lack responses to both IL-4 and IL-13 but the IL-13 produced in this strain can be modulated by IL-13Rα2. IL-13^−/−^/IL-4Rα^−/−^ mice have a complete absence of IL-4 and IL-13 responses. In STAT-6^−/−^ mice, only STAT-6-independent GATA3 expression and responses can occur.

### Cell lines and cell cultures

BS-C-1, an African green monkey kidney epithelial cell line, CV-1, an African green monkey kidney fibroblast cell line, P-815 (H-2^d^), a DBA/2 mouse-derived mastocytoma line and YAC-1 (H-2^a^), a Moloney murine leukemia virus-induced lymphoma line from A/Sn mouse, were obtained from American Type Culture Collection (Rockville, MD). All cells were maintained in Eagle’s Minimum Essential Medium supplemented with 10% fetal calf serum, 2mM L-glutamine, 120μg/ml penicillin and 200μg/ml streptomycin and neomycin sulfate.

### Viruses and infection

WT ECTV-Moscow strain [30], designated ECTV-WT, and a vIFN-γbp deletion mutant virus, designated ECTV-IFN-γbp^Δ^ [17] were used in this study. All ECTV stocks were propagated in BS-C-1 cells and titers determined by viral plaque assay (VPA) [31]. In all experiments, mice were inoculated subcutaneously (s.c.) with 10^3^ PFU of virus in the flank of the left hind limb (hock) under avertin anesthesia. This dose of virus was used in order to make meaningful comparisons with previous studies [8,25].

### Synthetic Peptides

ECTV-derived CD8 T cell peptide determinants [32] used in this study are shown in S1 Table. Peptides were synthesized at the Biomolecular Resource Facility, John Curtin School of Medical Research. They were purified via reverse-phase HPLC and the quality checked by mass spectroscopy. Peptides were stored in stock solutions at 10mM in 99.99% dimethyl sulfoxide (Sigma-Aldrich Inc. St. Louis, MO USA) and diluted in culture medium to the required concentration for use in the cellular assays.

### NK cell and CTL assays

The standard ^51^Chromium release assay [8] was used to determine *ex vivo* anti-ECTV CTL activity, using ECTV-infected and uninfected P815 (H-2^d^) target cells. YAC-1 cells were used as targets for splenic NK cell cytotoxicity assays. The fold change in cytolytic activity of GKO splenocytes compared with WT splenocytes shown in the results is based on % specific lysis at a given effector: target ratio. As an example, in the data shown on CTL responses, the % specific lysis mediated by IL-13^−/−^/IL-4Rα^−/−^ CTL at 75:1 effector: ratio is comparable to % specific lysis mediated by WT cells at 25:1 effector: ratio. That is, at a 3-fold lower number of splenocytes, the cytolytic activity of WT cells is comparable to that of IL-13^−/−^/IL-4Rα^−/−^ cells. Similarly, the % specific lysis mediated by IL-13^−/−^ cells at 75:1 effector: ratio is comparable to % specific lysis mediated by WT cells at 2.8:1 effector: ratio, i.e. a 27-fold lower number of WT splenocytes were required to mediate comparable levels of cytolytic activity mediated by IL-13^−/−^ cells.

### Flow cytometry

Total and granzyme B (GzB) expressing NK cells were quantified by flow cytometry. We used anti-CD49b-PE (clone DX5) to stain NK cells and anti-CD49b (DX-5)-PE plus anti-granzyme B-APC (clone MHGB05) to stain GzB expressing NK cells. Cells were also stained with anti-CD3-FITC (clone 17A2) to exclude CD3^+^ NKT cells in the analysis.

Total ECTV-specific or ECTV determinant-specific IFN-γ producing CD8 T cells were enumerated using intracellular cytokine staining. The protocol for detection of ECTV-specific CD8 T cells has been described previously [17]. Briefly, 10^6^ splenocytes were incubated with either 2x10^5^ virus-infected P815 cells or with synthetic peptides (S1 Table) at final concentrations of 0.1μM (K^d^-EVMA52_65–73_), 1 μM (L^d^-EVM026_26–34_) or 10μM (D^d^-EVM043_140–148_). After 2 hr, 0.1mg/ml brefeldin A was added and cells incubated for a further 3 hr before staining with anti-CD8α-APC (clone 53–6.7; BD Biosciences) and anti-IFN-γ- phycoerythrin (PE) (clone XMG1.2; BD Biosciences). Two hundred thousand total events were acquired on BD LSR Fortessa cell analyzer (BD Biosciences) and analyzed using FlowJo software (Tree Star Inc.). Antigen-specific IFN-γ production was calculated by subtracting the background values obtained with non-specific peptide (Herpes simplex virus-1 gB_498–505_) stimulation.

For enumeration of IFN-γ or IL-4 producing CD4 T cells, splenocytes from virus-infected mice were incubated at 37°C with media (unstimulated) or stimulated with Phorbol 12-Myristate 13-Acetate (PMA) plus Ionomycin (Calbiochem) (PMA: 0.1μg/ml; Ionomycin: 1μg/ml) for 5h as positive control. Following incubation, cells were stained with anti-CD4 (clone GK1.5), permeabilized for 20 min followed by intracellular staining with anti-IL-4 (clone 11B11) or anti-IFN-γ (clone XMG1.2) in staining buffer containing 0.1% saponin. Total events for cells were acquired using a BD LSR Fortessa cell analyzer and analyzed using FlowJo software (Tree Star Inc.). Antibodies were purchased from BD Biosciences, Biolegend and Invitrogen.

To estimate numbers of T-bet- or GATA-3-expressing CD4 T cells, 2 x10^6^ spleen cells were stained without re-stimulation with anti-CD3 and anti-CD4 in a 50μl volume for 30 mins at 4°C. The cells were then washed and re-suspended in 100μl of freshly prepared fix/perm buffer (FoxP3 transcription factor staining buffer kit, e-Bioscience) and incubated for 30 mins at 4°C. The cells were then washed twice with permeablization buffer and then incubated for 40 mins at 4°C with anti-GATA3 (Clone L50–823; BD Biosciences) and anti-T-bet (Clone 4B10, Biolegend) antibodies. The cells were washed twice with permeablization buffer and resuspended in buffer just prior to analysis on BD LSR Fortessa cell analyzer.

Cell numbers in experiments were determined by multiplying the percentage of cells positive for GzmB, IFN-γ (S1, S2, S3 Figs.), IL-4 (S4 Fig.), T-bet or GATA-3 by the total number of spleen cells obtained from each mouse.

### Determination of virus titers in tissues and blood

Organ tissue and blood were removed aseptically from infected mice and stored at −80°C until processed. Virus titers, expressed as log_10_ PFU/gram tissue or log_10_ PFU/100μl blood, were determined on BS-C-1 monolayers using the conventional VPA, as described previously [9].

### Statistical analysis

Statistical analyses of experimental data, employing parametric and nonparametric tests as indicated in figure legends, were performed using GraphPad Prism (GraphPad Software, San Diego, USA). The Kaplan-Meier Log rank test was used to determine p values for survival proportions. Two-way ANOVA followed by Fisher’s Least Significant Difference (LSD) test was used to determine significance for viral load, NK, CD8 and CD4 T cell responses.

## Results

### Deficiencies in Th2 cytokine signaling exacerbate ECTV infection

**Fig 1:**
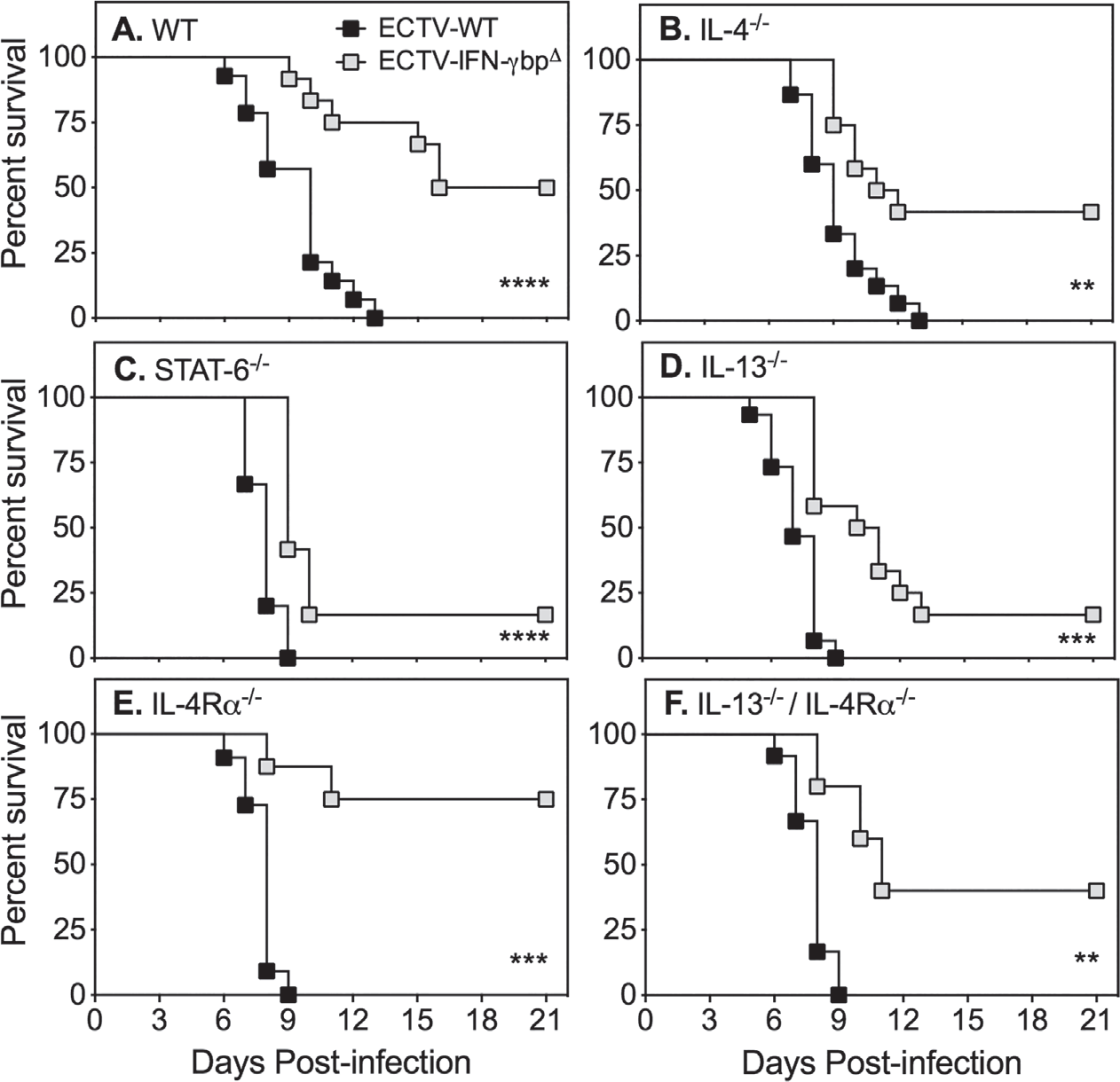
Response of WT and GKO BALB/c strains to infection with ECTV-WT or ECTV-IFN-γbp^Δ^. Groups of female mice were infected with ECTV-WT or ECTV-IFN-γbp^Δ^. Mice were monitored daily for 21 days for disease signs. Data shown are combined results obtained from two separate experiments in which 5–15 mice per strain were used (S2 Table). *P* values for survival proportions were obtained by using Kaplan-Meier Log rank statistical test: **, *p* < 0.01; ***, *p* < 0.001; ****, *p* < 0.0001. The survival rates of WT mice compared to GKO mice infected with WT or mutant virus are presented in S5 and S6 Figs., respectively and the statistical analysis presented in S2 Table.

Groups of WT and GKO mice were infected with ECTV-WT or ECTV-IFN-γbp^Δ^ and monitored for 21 days to determine whether the absence or presence of vIFN-γbp during ECTV infection in BALB/c mice lacking IL-4, IL-13, IL-4Rα, STAT-6 or both IL-13 and IL-4Rα may result in better recovery rates. We compared the outcome of infection with the 2 viruses in individual mouse strains (Fig. 1), ECTV-WT infection in WT versus individual GKO mouse strains (S5 Fig.) and ECTV-IFN-γbp^Δ^ infection of WT versus individual GKO mouse strains (S6 Fig.). The detailed statistical analysis is presented in S2 Table.

Regardless of genotype, all groups of mice infected with ECTV-WT succumbed to mousepox with 100% mortality (Fig. 1, S5 Fig., S2 Table). Notably, most GKO strains succumbed more rapidly than WT mice to ECTV-WT infection. Absence of one or more factors associated with Th2 response development did not provide any added advantage to hosts infected with WT virus. In contrast, all groups of mice infected with ECTV-IFN-γbp^Δ^ had better rates of recovery compared with ECTV-WT infection (Fig. 1, S6 Fig., S2 Table). However, even despite the improved recovery rates compared with WT virus infection, some GKO strains infected with mutant virus succumbed to mousepox significantly more rapidly than WT mice. In particular, mice deficient in IL-13 or STAT-6 had the lowest recovery rates and succumbed to disease more rapidly than WT mice infected with ECTV-IFN-γbp^Δ^. These experiments established that absence of vIFN-γbp significantly assisted in the recovery of WT mice from ECTV infection but did not improve recovery of the GKO strains. They also revealed that absence of IL-13, IL-4Rα, STAT-6 or both IL-13 and IL-4Rα increased susceptibility of mice to ECTV-WT infection.

### Lack of consistent correlation between increased susceptibility and viral load

Susceptibility to ECTV is associated with uncontrolled virus replication, particularly in the liver and spleen [5–8]. We measured titers of ECTV-WT and ECTV-IFN-γbp^Δ^ in livers, spleens and blood collected from animals at day 7 post-infection (p.i.) to determine whether the increased susceptibility of some strains was due to increased virus load. This time point was chosen as WT BALB/c mice infected with ECTV-WT begin to succumb to mousepox from day 7 p.i. as a result of high viral load [8,17].

**Fig 2:**
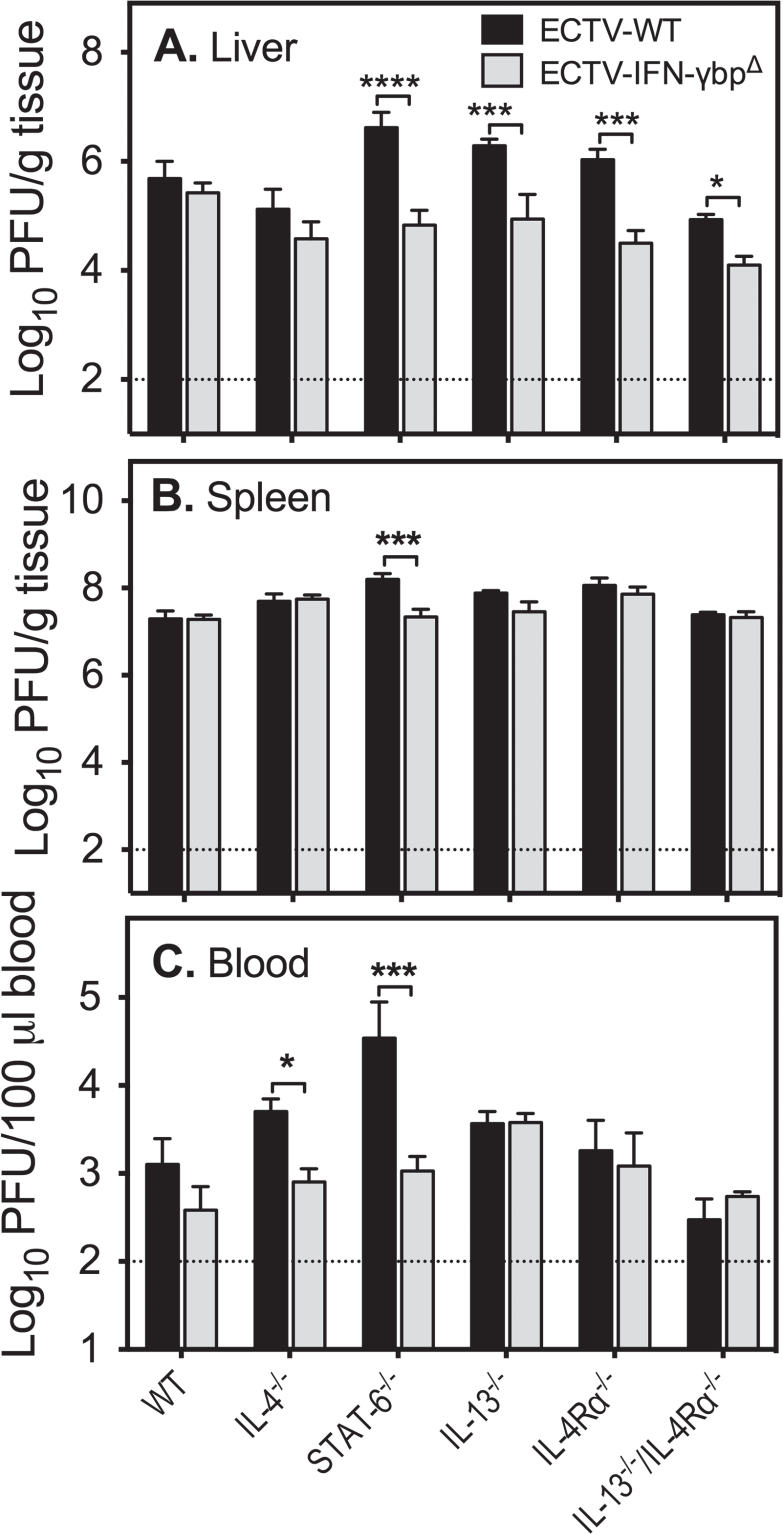
Viral load in organs and blood of WT and GKO mice infected with ECTV-WT or ECTV-IFN-γbp^Δ^. Groups of 5 mice were infected with either ECTV-WT or ECTV-IFN-γbp^Δ^ and sacrificed at day 7 p.i. Viral load was determined by viral plaque assay and expressed as Log_10_ PFU virus/gram tissue. The dotted line indicates the limit of virus detection by viral plaque assay. Two-way ANOVA followed by Fisher’s LSD test for significance between (i) ECTV-WT titers in WT mice compared with all strains, (ii) ECTV-IFN-γbp^Δ^ load in WT mice compared with all strains, and (iii) ECTV-WT compared with ECTV-IFN-γbp^Δ^ load in individual strains (A) liver, (B) spleen, and (C) blood. The detailed statistical analysis of WT and mutant virus titers in each of the 5 GKO mouse strains is presented in S3 (Liver), S4 (Spleen) and S5 (Blood) Tables. Asterisks indicate significant differences in organ titers comparing ECTV-WT or ECTV-IFN-γbp^Δ^. ****, p< 0.0001; ***, 0.0001< p <0.001; **, 0.001< p <0.01; * 0.01< p <0.05.

In the liver, ECTV-WT replicated to significantly higher levels in STAT-6^−/−^ mice compared to WT animals but titers were otherwise comparable across other strains (Fig. 2, S3 Table). ECTV-IFN-γbp^Δ^ titers on the other hand were significantly lower in IL-4^−/−^ and IL-13^−/−^/IL-4Rα^−/−^ mice compared to WT mice but no other differences were evident. A comparison of ECTV-WT and ECTV-IFN-γbp^Δ^ titers indicated that the mutant virus replicated at significantly lower levels in STAT-6^−/−^, IL-13^−/−^, IL4-Rα^−/−^, and IL-13^−/−^/IL-4Rα^−/−^ mice (S3 Table).

In the spleen, the WT viral load increased significantly in STAT-6^−/−^, IL-13^−/−^, and IL-4Rα^−/−^ mice compared with WT animals (Fig. 2, S4 Table). On the other hand, ECTV-IFN-γbp^Δ^ titers were significantly higher in IL-4^−/−^ and IL-4Rα^−/−^ mice compared with WT mice. In a comparison of WT and mutant virus titers among all mouse strains, it was apparent that ECTV-IFN-γbp^Δ^ replicated to lower levels only in the spleen of STAT-6^−/−^ mice (S4 Table).

Finally, in the blood, ECTV-WT titers were increased in STAT-6^−/−^ mice compared to WT mice (Fig. 2, S5 Table) but no differences in its replicative capacity were evident in the other strains. ECTV-IFN-γbp^Δ^ replicated to higher levels in IL-13^−/−^ mice compared to WT animals but titers were lower than that of WT virus in STAT-6^−/−^ and IL-4^−/−^ mice.

Taken together, with the exception of STAT-6^−/−^ mice in which ECTV-WT titers were significantly increased in all organs compared to WT mice, there was no consistent correlation between increased susceptibility of individual mouse strains and viral load in visceral organs or blood.

### The effect of vIFN-γbp on NK cell responses in GKO mice with deficiencies in Th2 cytokine signaling

NK cells play an important role in virus control during the acute phase of ECTV infection and recovery of mice [5,17,33,34]. We measured the splenic NK cell activity at the peak of the response at day 5 p.i. in order to determine whether the absence of vIFN-γbp and/or factors associated with Th2 responses augmented these responses following infection.

**Fig 3:**
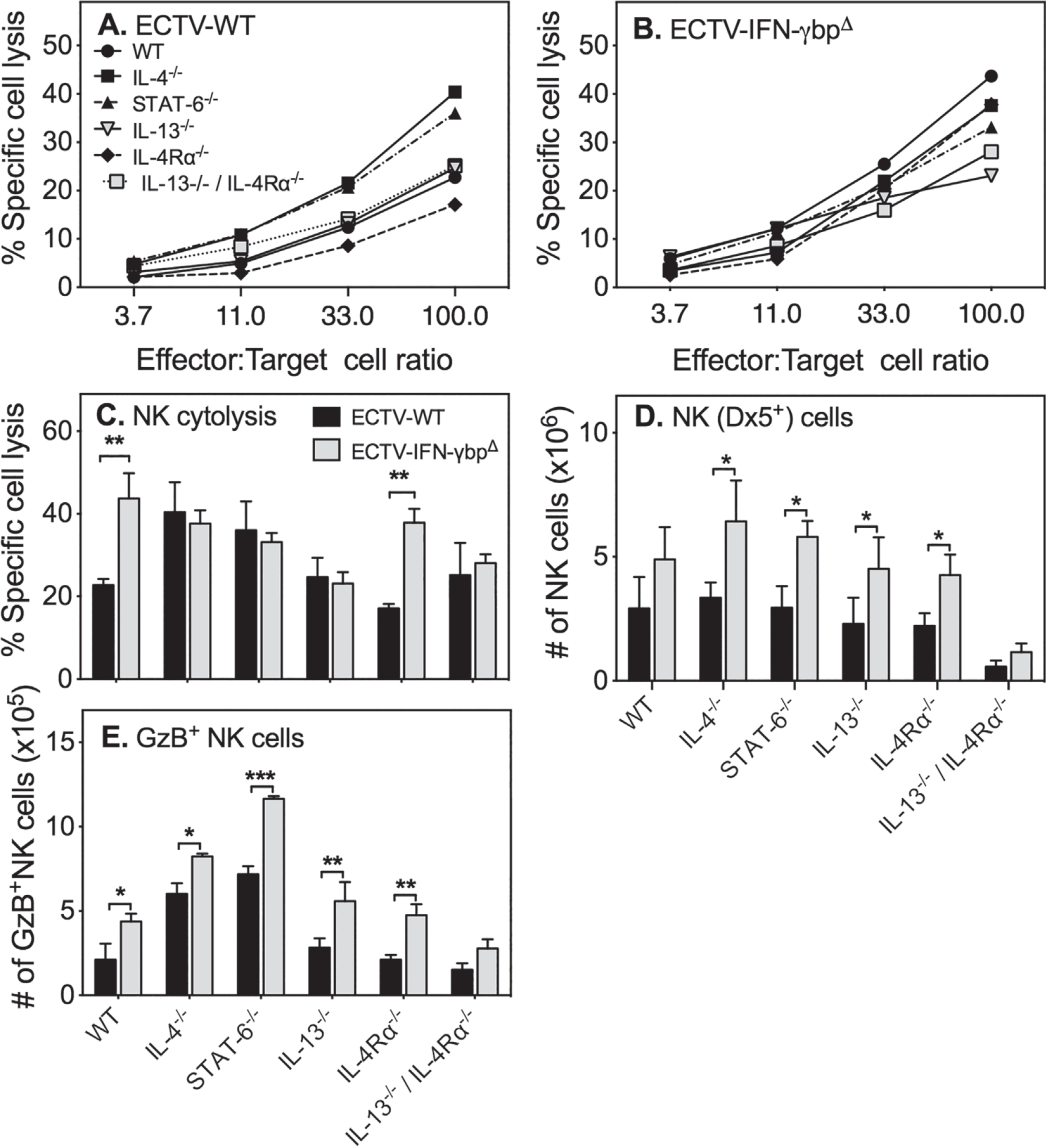
NK cell responses to ECTV-WT and ECTV-IFN-γbp^Δ^. Groups of 5 mice were infected with ECTV-WT or ECTV-IFN-γbp^Δ^, sacrificed 5 days later and the cytolytic activities of splenic NK cells measured. Data shown are % specific lysis of ^51^Cr-labelled YAC-1 target cells by splenic NK cells from (A) ECTV-WT-infected or (B) ECTV-IFN-γbp^Δ^-infected WT and GKO mouse strains. (C) Percent specific lysis of targets by splenocytes from the various strains infected with ECTV-WT or ECTV-IFN-γbp^Δ^ at 100:1 effector-to-target ratio. (D) Numbers of GzB^+^ NK cells. Data shown are means SEM of results obtained from one of 3 separate experiments. Statistical significance was determined by 2-way ANOVA followed by Fisher’s LSD test for significance between groups. For panel C, (i) NK cell responses to ECTV-WT vs. ECTV-IFN-γbp^Δ^ in WT mice (*p* = 0.0024) and IL-4Rα^−/−^ mice (*p* < 0.0023); (ii) NK cell responses to ECTV-WT in WT mice vs IL-4^−/−^ (*p* = 0.009), WT mice vs STAT-6^−/−^ mice (**p = 0.0476); (iii) NK cell response to ECTV-IFN-γbp^Δ^ in WT vs IL-13^−/−^ (*p* = 0.0024), WT vs IL-13^−/−^/IL-4Rα^−/−^ mice (*p = 0.02). For panel D, (i) GzB^+^ NK cell numbers in ECTV-WT vs. ECTV-IFN-γbp^Δ^ infected strains: WT mice (*p* = 0.02), IL-4^−/−^ mice (*p* = 0.02), STAT-6^−/−^(*p* = 0.0002), IL-13^−/−^ (p = 0.0068) and IL-13^−/−^/IL-4Rα^−/−^ mice (*p* = 0.008); (ii) GzB^+^ NK cell numbers in ECTV-WT infected WT mice vs IL-4^−/−^ mice (p = 0.0006) and WT mice vs STAT-6^−/−^ mice (p<0.0001); (iii) GzB^+^ NK cell numbers in ECTV-IFN-γbp^Δ^ infected WT mice vs IL-4^−/−^ mice (p = 0.0007) and WT mice vs STAT-6^−/−^ mice (p<0.0001). *, *p* < 0.05; **, *p* < 0.01; ***, *p* < 0.001.

ECTV-WT induced weak splenic NK cell activity in WT, IL-13^−/−^, IL-4Rα^−/−^ and IL-13^−/−^/IL-4Rα^−/−^ mice (Fig. 3A). The only exceptions were the IL-4^−/−^ and STAT-6^−/−^ strains in which the responses were about 3-fold higher than in WT animals. ECTV-IFN-γbp^Δ^ induced a 3-fold higher NK cell cytolytic activity compared with ECTV-WT in WT and IL-4Rα^−/−^ mice but did not affect responses in the other strains (Fig. 3B). The magnitude of NK cell responses to both mutant and WT viruses were similar in IL-4^−/−^, IL-13^−/−^, STAT-6^−/−^ and IL-13^−/−^/IL-4Rα^−/−^ mice. For the purpose of comparison, the NK cell responses to both WT and mutant mouse strains at 100: 1 effector to target cell ratio is shown in Fig. 3C.

The total numbers of DX5^+^ NK cells were not altered significantly in GKO strains compared with WT mice following infection with ECTV-WT, with the exception of the IL-13^−/−^/IL-4Rα^−/−^ mice in which the numbers were about 5-fold lower (Fig. 3D). Numbers of NK cells increased significantly in most GKO strains following infection with the mutant virus except for WT and IL-13^−/−^/IL-4Rα^−/−^ mice. We also assessed the expression of granzyme B (GzB), a serine protease found in granules of cytotoxic lymphocytes, to determine whether there was any association between the cytolytic activity of NK cells and GzB expression. Flow cytometric analysis revealed that WT virus infection increased GzB^+^ NK cell (CD3-DX5^+^) numbers in IL-4^−/−^ and STAT-6^−/−^ mice compared to WT mice (Fig. 3E). In contrast, mutant virus infection increased numbers of GzB^+^ cells in all strains compared to WT virus. Nonetheless, increases in GzB^+^ cell numbers did not always result in a corresponding increase in cytolytic activity (Fig. 3A-3C). As an example, infection of STAT-6^−/−^ mice with ECTV-WT resulted in increased GzB^+^ cell numbers over and above those in WT mice and infection with the mutant virus further increased numbers. However, regardless of the type of virus used, the cytolytic activities of NK cells in WT and STAT-6^−/−^ mice were comparable (Fig. 3A-3C).

### Varied CD8 T cell responses in mice lacking Th2 cytokine signaling is differentially regulated by vIFN-γbp

CD8 T cells are an important source of IFN-γ production in mousepox beginning at day 6 p.i. The levels of IFN-γ produced by these cells closely parallel the magnitude of their cytolytic activity [8] and both responses peak between days 7–8 p.i. We assessed whether deletion of vIFN-γbp augmented IFN-γ production and CTL activity in CD8 T cells in WT and GKO mice at day 7 p.i.

**Fig 4:**
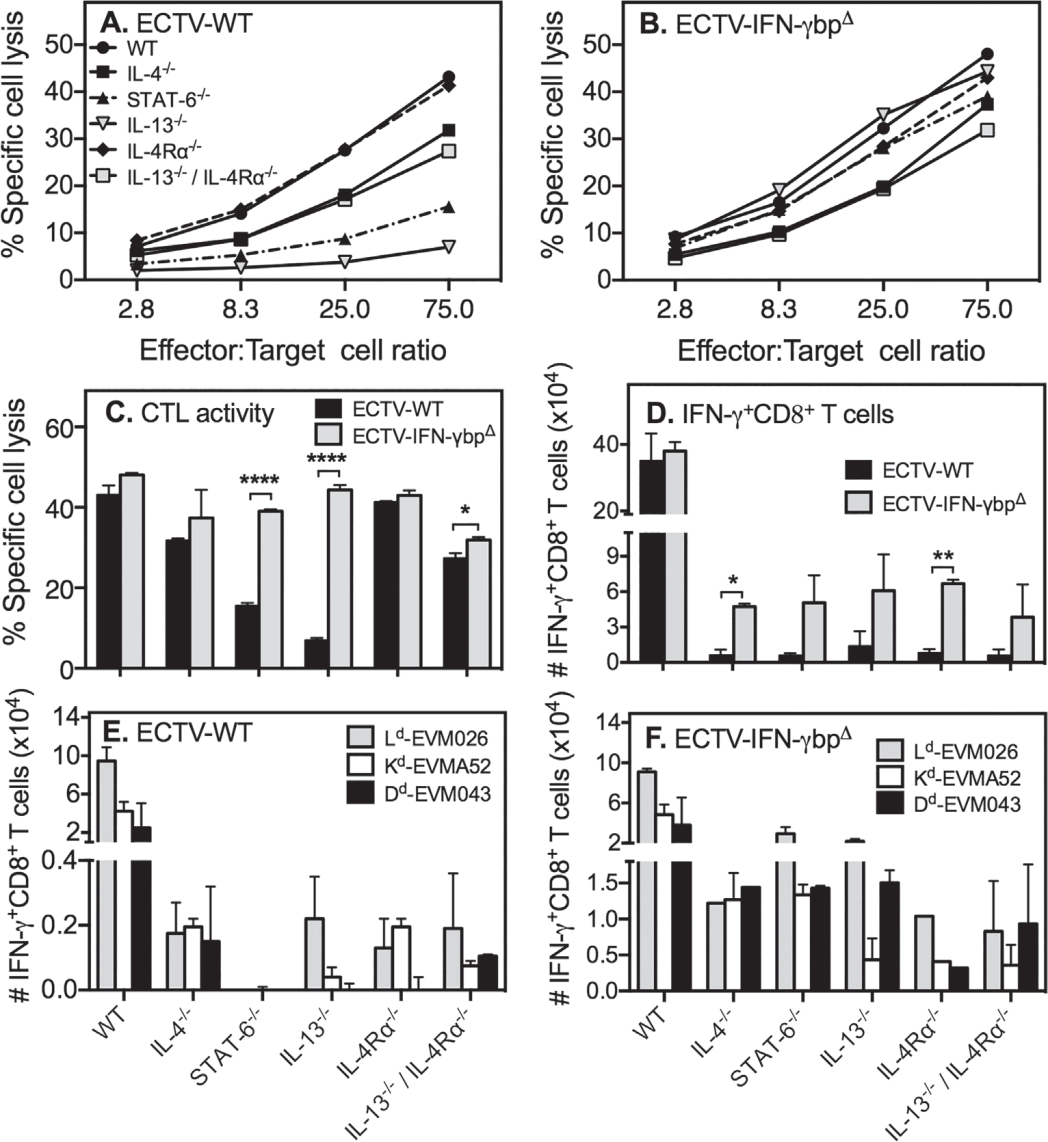
CD8 T cell responses to ECTV-WT and ECTV-IFN-γbp^Δ^. Mice (n = 5–10/group) were infected with ECTV-WT or ECTV-IFN-γbp and sacrificed 7 days later to measure splenic CTL activity. Shown is % specific lysis of ^51^Cr-labelled, ECTV-infected P815 target cells by splenocytes from (A) ECTV-WT- or (B) ECTV-IFN-γbp^Δ^-infected WT and GKO mice. (C) Percent specific lysis of targets by splenocytes at 75:1 effector-to-target ratio. Two-way ANOVA followed by Fisher’s LSD test for significance was used for statistical analysis: (i) CTL response to ECTV-WT vs. ECTV-IFN-γbp^Δ^ in IL-13^−/−^ (p < 0.0001) and STAT-6^−/−^ (p < 0.0001) (ii) CTL response to ECTV-WT in WT vs IL-4^−/−^ (p = 0.0083), vs STAT-6^−/−^ (p = 0.0003), vs IL-13^−/−^ (p = 0.0001), vs IL-13^−/−^/IL-4Rα^−/−^ (p = 0.0037) mice; (iii) CTL response to ECTV-IFN-γbp in WT vs STAT-6^−/−^ (p = 0.0002); vs IL-13^−/−^ (p = 0.0478); vs IL-4Rα^−/−^ (p = 0.0174) and IL-13^−/−^/IL-4Rα^−/−^ (p = 0.000046) mice. ****, p<0.0001. (D—F) Spleen cells from mice infected mice were re-stimulated for 5 h with ECTV-infected P815 cells or H-2^d^ 8 T cell peptide determinants prior to intracellular IFN-γ staining followed by flow cytometry. Data shown are means of absolute numbers of ECTV-specific IFN-γ^+^ 8 T cells in spleens of ECTV-WT- vs. ECTV-IFN-γbp^**Δ**^-infected mice (D) and determinant-specific IFN-γ^+^ 8 T cells following infection with ECTV-WT (E) or ECTV-IFN-γbp (F) viruses. Two-way ANOVA followed by Fisher’s LSD test for significance was used for statistical analysis. For panels D, E and F, IFN-γ^+^ 8 T cell numbers in WT mice were significantly higher (p<0.0001) than numbers in all GKO strains. Data shown for D-F are representative of one of three independent experiments with similar results. *, *p*< 0.05; **, *p* < 0.01; ***, *p* < 0.0001.

Absence of IL-4Rα signaling did not affect CTL responses since WT and IL-4Rα^−/−^ mice infected with ECTV-WT generated similar levels of killing (Fig. 4A). The responses in IL-4^−/−^ and IL-13^−/−^/IL-4Rα^−/−^ mice were about 3-fold lower than WT mice. However, the most striking differences were evident in STAT-6^−/−^ and IL-13^−/−^ mice in which the responses were about 9-fold and 27-fold lower, respectively, compared to responses in WT mice. The significant reduction in CTL response in IL-13^−/−^ mice was partially reversed when IL-4Rα was also absent. The data indicated that IL-4, STAT-6 and IL-13, which are normally associated with development of Th2 responses, play important and non-redundant roles in the generation of optimal antiviral CTL responses.

The vIFN-γbp had minimal impact on CTL activity in WT, IL-4^−/−^ and IL-4Rα^−/−^ mice as these strains generated comparable responses to WT or mutant virus infection (Fig. 4A-4C). The most dramatic effects of infection with ECTV-IFN-γbp^Δ^ in the GKO mouse strains were evident in STAT-6^−/−^ and IL-13^−/−^ mice. These groups had significantly diminished CTL responses to WT virus infection (Fig. 4A and 4C). However, mutant virus infection in both mouse strains induced robust CTL activities, which were almost as high as the magnitude in WT mice (Fig. 4B and 4C). Thus, vIFN-γbp had a greater deleterious effect on CD8 T responses in STAT-6^−/−^ and IL-13^−/−^ mice compared with the other GKO strains.

ECTV-WT induced significantly higher (p<0.0001) numbers of virus-specific IFN-γ-producing CD8 T cells in WT mice compared to all GKO strains, in which numbers were reduced by 20–40-fold (Fig. 4D). While ECTV-IFN-γbp^Δ^ induced comparable numbers of IFN-γ-producing CD8 T cells as WT virus in WT mice, absence of vIFN-γbp only marginally increased numbers of IFN-γ^+^ CD8 cells in each GKO strain (Fig. 4D).

The MHC class I L^d^-restricted response generally dominates at day 7 p.i. in BALB/c mice. Numbers of L^d^-, K^d^- and D^d^-restricted determinant-specific IFN-γ^+^ cells were significantly higher (p<0.0001) in WT mice infected with ECTV-WT compared to all GKO strains (Fig. 4E). The reduction in virus-specific IFN-γ-producing CD8 T cell numbers seen in GKO strains (Fig. 4D) was clearly reflected in the determinant-specific responses, which were nonetheless varied (Fig. 4E). Curiously, there was a complete lack of reactivity to the three H-2d-restricted viral determinants in the STAT-6^−/−^ strain. Although we do not have an explanation for this finding, it nonetheless suggested the possibility that the response might have been directed against other H-2d determinants.

In WT mice, ECTV-IFN-γbp^Δ^ infection generated determinant-specific IFN-γ^+^ CD8 T cells (Fig. 4F) that were comparable in numbers to ECTV-WT infection. Mutant virus infection increased numbers of determinant-specific IFN-γ^+^ CD8 T cells in all GKO strains (Fig. 4F), over and above those induced by ECTV-WT (Fig. 4E). In particular, the biggest increases were seen in STAT-6^−/−^ mice and IL-13^−/−^ mice (Fig. 4F). The mechanism(s) though which absence of vIFN-γbp at least partially reconstituted the determinant-specific IFN-γ^+^ CD8 T cell responses in STAT-6^−/−^ mice or increased both the total (Fig. 4D) and determinant-specific responses (Fig. 4F) in the other GKO strains is currently unknown but merits investigation.

### Effect of vIFN-γbp on IFN-γ or IL-4 production by CD4 T cells deficient in Th2 cytokine signaling

The preceding data established that deficiencies in specific Th2 cytokine signaling significantly reduced the numbers of IFN-γ^+^ CD8 T cells and that vIFN-γbp partially modulated the response. It was therefore of interest to determine whether the combined deficiencies in Th2 cytokines and vIFN-γbp promoted Th1 responses in CD4 T cells.

**Fig 5:**
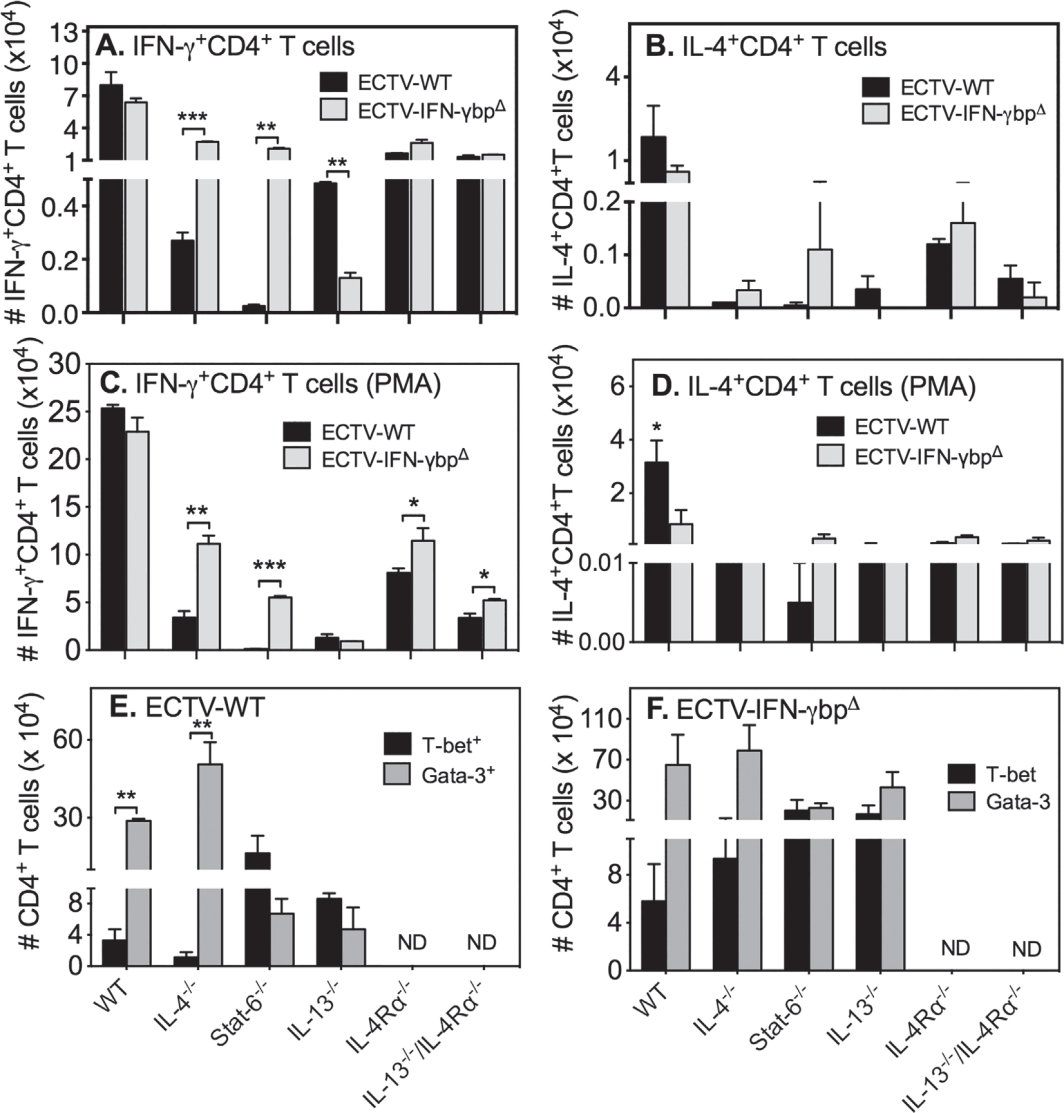
Numbers of CD4 T cells expressing IFN-γ, IL-4, T-bet or GATA-3. Mice (n = 5–10/group) were infected with ECTV-WT or ECTV-IFN-γbp^Δ^, sacrificed on day 7 p.i. and splenocytes used for intracellular staining for IFN-γ, IL-4, T-bet or GATA-3 without stimulation (A and B) or with PMA + ionomycin stimulation (C and D). Shown are means of absolute numbers of IFN-γ^+^CD4 T cells with no stimulation (A) or PMA + ionomycin stimulation (C), IL-4^+^CD4 T cells with no stimulation (B) or PMA + ionomycin stimulation (D). Also shown are means of absolute numbers of T-bet^+^ and GATA-3^+^ CD4 T cells following ECTV-WT (E) or ECTV-IFN-γbp^Δ^ (F) infection. Two-way ANOVA followed by Fisher’s LSD test for significance was used. For panel A, numbers of IFN-γ^+^CD4 T cells in WT mice for both viruses were significantly higher (p<0.0001) compared to numbers in all GKO strains. In IL-4^−/−^ and STAT-6^−/−^ mice, IFN-γ^+^CD4 T cell numbers generated by ECTV-IFN-γbp^Δ^ infection were significantly higher (*p* = 0.0005 and *p* = 0.0013, respectively) compared to WT virus infection. In IL-13^−/−^ mice, IFN-γ^+^CD4 T cell numbers generated by WT virus infection were significantly higher (*p* = 0.0034) compared to ECTV-IFN-γbp^Δ^ infection. For panel B, numbers of IL-4^+^CD4 T cells in WT mice for both viruses were significantly higher (p<0.01) compared to numbers in all GKO strains. No other significant differences were found. For panel C, numbers of IFN-γ^+^CD4 T cells in WT mice for both viruses were significantly higher (p<0.0001 in ECTV-WT and *p* < 0.001 in ECTV-IFN-γbp^Δ^) compared to numbers in all GKO strains. In IL-4^−/−^, STAT-6^−/−^ IL-4Rα^−/−^ and IL-13^−/−^ / IL-4Rα^−/−^ mice, IFN-γ^+^CD4 T cell numbers generated by ECTV-IFN-γbp^Δ^ infection were significantly higher compared to WT virus infection. For panel D, numbers of IL-4^+^CD4 T cells in WT mice for ECTV-WT virus was significantly higher (p<0.05) compared to numbers in all GKO strains. In WT mice, IL-4^+^CD4 T cell numbers generated by WT virus infection were significantly higher (*p<*0.05) compared to ECTV-IFN-γbp^Δ^ infection. For panel E, T-bet^+^ CD4 T cell numbers were significantly increased (p<0.01) in STAT-6^−/−^ and IL-13^−/−^ mice compared to WT animals. For panel F, no significant differences were found between strains. Data shown for panels A and B are from one of two independent experiments with similar results. Data shown for panels C-F are from one experiment. *, *p* < 0.05; **, *p* < 0.01; ***, *p* < 0.0001.

Intriguingly, WT mice infected with ECTV-WT or ECTV-IFN-γbp^Δ^ generated the highest numbers of IFN-γ^+^ CD4 T cells whereas the numbers in all GKO strains were significantly (p<0.0001) reduced (Fig. 5A). Of note, STAT-6^−/−^ mice infected with WT virus had the lowest number of IFN-γ producing CD4 T cells but it was the only strain in which mutant virus infection increased numbers significantly by nearly 90-fold. Similarly, in the case of IL-4 production, WT mice infected with WT or mutant virus generated the largest number of IL-4^+^ CD4 T cells but numbers were significantly lower (p<0.01) in all GKO strains of mice (Fig. 5B). Data shown in Fig. 5A and 5B are using non-stimulated cells. CD4 T cells from IL-4^−/−^ mice did not produce IL-4 as expected but IL-4^+^ CD4 T cell numbers were also minimal in STAT-6^−/−^ and IL-13^−/−^ mice. *In vitro* stimulation of splenocytes with PMA + ionomycin increased the numbers of IFN-γ (Fig. 5C) and IL-4 (Fig. 5D) producing CD4 T cells in all groups but the response in WT cells was still much higher than in GKO cells.

We interrogated CD4 T cells by flow cytometry to detect expression of intracellular T-bet and GATA-3, the signature Th1 and Th2 transcription factors, respectively. In WT mice infected with ECTV-WT, CD4 T cells were predominantly GATA-3^+^ (Fig. 5E). IL-4 deficiency further increased GATA-3^+^ CD4 T cell numbers whereas STAT-6 or IL-13 deficiency increased T-bet^+^ cell numbers over and above those in WT mice. On the other hand, ECTV-IFN-γbp^Δ^ infection did not significantly change T-bet^+^ or GATA-3^+^ cell numbers in GKO strains compared to those in WT mice. However, in comparing numbers induced by ECTV-WT infection (Fig. 5C), both T-bet^+^ and GATA-3^+^ cell numbers were increased in all strains following infection with ECTV-IFN-γbp^Δ^ (Fig. 5D). Increases in T-bet^+^ cell numbers did not necessarily translate to increased numbers of IFN-γ^+^ CD4 T cells (Fig. 5A and 5C) and likewise, increases in GATA-3^+^ cells did not result in increases in IL-4^+^ CD4 T cells (Fig. 5, panels B and D). On the contrary, deficiency in specific Th2 cytokines, transcription factor or cytokine receptor resulted in reduced numbers of IFN-γ^+^ and IL-4^+^ CD4 T cells. vIFN-γbp modulated the IFN-γ response only in STAT-6^−/−^ mice but that did not assist in the recovery of this strain from mutant virus infection. Deficiency in Th2 cytokine signaling did not bias CD4 T cells towards a Th1 response.

## Discussion

A type 1/Th1-like cytokine response coupled with robust cell-mediated immunity is critical for recovery from mousepox whereas a type 2/Th2-like response associated with an absent or weak IFN-γ response and cell-mediated immunity is associated with susceptibility [8]. Deficiency in IFN-γ [9,10] or its suppression through over-expression of IL-4 by recombinant ECTV in resistant mice results in generation of poor cell-mediated immunity, overcomes resistance to mousepox and results in fulminant disease [11,12]. While it may be speculated that type 1-like responses were important for recovery of humans from smallpox, there is circumstantial evidence that Th2-biased responses can significantly exacerbate the disease and result in increased mortality.

In non-vaccinated humans, about 90% of cases were of the ordinary-type smallpox with a case fatality rate of about 30–40% [35]. A more severe form of the disease, hemorrhagic-type smallpox, characterized by mucosal and skin hemorrhage, was mainly seen in pregnant women. Early hemorrhagic-type smallpox was associated with almost 100% mortality regardless of vaccination status and pregnant women with smallpox had 3–4 times higher case fatality rates than non-pregnant women and men of the same age groups [35,36]. Although the reasons for higher case fatality rates were not known at the time when smallpox was endemic, it is now recognized that an immune bias towards a Th2-like response associated with pregnancy [37–39] may have been responsible. That Th2-biased response can exacerbate smallpox even in vaccinated individuals is significant. In this regard, the demonstration that infection of previously vaccinated mice with recombinant ECTV encoding IL-4 can completely overcome protective immunity and genetic resistance [11,12] helped establish that over exuberant Th2-biased responses increase susceptibility to OPV infections. This raises the question of whether defective or sub-optimal Th2 responses allow for the generation of more effective antiviral immunity. Our results indicate they do not.

Deficiencies in IL-4, STAT-6, IL-13 and/or IL-4Rα signaling on immune response generation were varied and further modulated by vIFN-γbp but 3 broad categories of outcomes were evident. In the first, the IL-4^−/−^ mice had comparable recovery rates to WT mice regardless of whether they were infected with WT or mutant virus. In the second, the IL-4Rα^−/−^ and IL-13^−/−^/IL-4Rα^−/−^ mice exhibited increased susceptibility to WT virus infection but the absence of vIFN-γbp during infection mitigated the increased susceptibility. Finally, mice deficient in IL-13 or STAT-6 were significantly more susceptible than WT animals in response to WT or mutant virus infection. Susceptibility to ECTV is attributed to uncontrolled generalized virus replication in visceral organs, including in the liver [6,7], spleen and blood [40]. However, in the current study, viral load in organs and blood 7 days after infection did not consistently correlate with increased susceptibility, with the exception of STAT-6^−/−^ mice. This outcome is surprising and might have been a consequence of the extent to which deficiencies in IL-4, IL-13 and/or IL-4Rα, combined with immunomodulation by vIFN-γbp impacted on inflammation, the immune response and virus control. Viral load in the liver and spleen generally increase exponentially over time if not effectively controlled by the immune response. However, the blood viral load is usually only increased following primary, secondary and subsequent occurrence of viremia following release of virions from the visceral organs. It is possible that kinetics of virus replication in the different GKO mouse strains was different and measurement of viral load at a single time-point might not have revealed the extent to which the viruses replicated and spread. However, the fact that most GKO mice succumbed to mousepox at or after 6 days post-infection suggests that measurement of viral titers at day 7 should have indicated if uncontrolled virus replication was the cause of death. Another finding of note is that deficiency in IL-13 or IL-4Rα resulted in comparable viral titers in liver, spleen and blood but titers were lower in the liver and blood in IL-13^−/−^/IL-4Rα^−/−^ mice. While the reason for this is unclear, it might be explained, at least in part by the CTL responses generated in the absence of IL-13 or both IL-13 and IL-4Rα (see below).

Absence of IL-4, STAT-6, IL-13 or IL-4Rα signaling clearly had a significant impact on both CD4 and CD8 T cell responses, which were modulated to some extent by vIFN-γbp. Contrary to our prediction, the numbers of IFN-γ^+^ CD4 and CD8 T cells as well as IL-4^+^ CD4 T cells were decreased in all GKO strains compared to WT mice. Absence of vIFN-γbp during infection had minimal effects on CD4 T cell responses with the exception of STAT-6^−/−^ mice in which mutant virus infection significantly increased IFN-γ^+^ CD4 T cell numbers. The most striking reductions in the anti-ECTV CTL responses were seen with WT virus infected STAT-6^−/−^ and IL-13^−/−^ mice, which were almost completely reversed when these strains were infected with ECTV-IFN-γbp^Δ^. Deficiency in the IL-4Rα alone did not have any impact on the CTL response but in the IL-13^−/−^ mice, the significant reduction in CTL activity was partially reversed when IL-4Rα was also absent. This is an interesting finding and although the mechanisms involved are not known, it suggests that in the absence of IL-13, IL-4 signaling through the IL-Rα was far more detrimental to induction of antiviral CTL responses than in the complete absence of IL-4 and IL-13 responses in IL-13^−/−^/IL-4Rα^−/−^ mice. We speculate that IL-13 might modulate the IL-4 response in induction of ECTV-specific CTL responses. Another important finding in the STAT-6^−/−^ mice is the complete lack of reactivity of CD8 T cells to any of the H-2d determinants that were tested. One explanation for the complete lack of reactivity to the three H-2^d^-restricted viral determinants in the STAT-6^−/−^ strain is that the response might have been directed against other determinants. It is also possible that results could be influenced by subtle changes in reactivity of cells maturing in a STAT-6-deficient environment. Such changes in immune responses may be due to altered peptide expression that might be negligible or difficult to identify or quantify, but should nonetheless be considered for investigations in our future studies. It is curious why Th2-response linked genes influence the reactivity of CD8 T responses but may be related, at least in part, to potential defects in antigen presentation by dendritic cells as discussed below.

Although IFN-γ impacts on the quality and magnitude of the anti-ECTV CTL response [8], it was evident that the augmented CTL activity in IL-13^−/−^ and STAT-6^−/−^ mice was not related to the marginally increased numbers of virus-specific IFN-γ^+^ CD8 cells, which occurred in all GKO strains. It is also possible that factors other than CD8 T cell-derived IFN-γ influenced the cytolytic activity of CTL in IL-13^−/−^ and STAT-6^−/−^ mice. As CD4 T cells are important for generation of optimal CTL responses to ECTV infection [41], we considered the possibility that IFN-γ-producing CD4 T cells may have impacted on the magnitude of CTL responses. While this might have been the case in STAT-6^−/−^ animals (Fig. 5A) in which numbers of IFN-γ^+^ CD4 cells substantially increased following infection with the mutant virus, it did not hold true for the IL-13^−/−^ mice. There is therefore little or no evidence for any consistent correlation between the numbers of IFN-γ^+^ CD4 T cells and the magnitude of the CD8 T cell-mediated CTL responses in individual mouse strains.

STAT-6-dependent IL-4 production has been purported to be associated with susceptibility of BALB/c mice to ECTV infection [25] but IL-4 deficiency in BALB/c mice has no impact on disease outcome [8]. Since IL-4^−/−^ mice can respond to IL-13, we hypothesized that the cytokine might compensate for absence of IL-4 to drive Th2-like responses. Our data established that this was not the case and that the responses to, and outcome of infection with, ECTV in IL-4^−/−^, IL-13^−/−^ and STAT-6^−/−^mice are very different. First, IL-13^−/−^ and STAT-6^−/−^ mice were far more susceptible to WT or mutant virus infection than IL-4^−/−^ mice. Second, the CTL response to WT virus was marginally reduced in IL-4^−/−^ mice compared to WT animals, but the effect was far more significant in IL-13^−/−^ and STAT-6^−/−^ mice. This is an important and novel finding in that factors normally associated with Th2 responses play critical and non-redundant roles in the generation of optimal antiviral CD8 CTL responses. The mechanism(s) through which IL-13 or STAT-6 impact on CTL responses generation during ECTV infection is currently unknown but merit a detailed investigation.

Naïve CD8^+^ T cells in IL-4^−/−^, IL-13^−/−^ and STAT6^−/−^ mice express lower levels of IL-4Rα making them less responsive to IL-4 and IL-13 during T cell priming [42]. It is therefore possible that in IL-13^−/−^ mice, IL-4 signaling alone through activation of STAT-6 may promote sufficient down-regulation of CD8 co-receptor densities on naïve CD8^+^ T cells following cognate antigen encounter during ECTV infection, and lessen the anti-viral capacity of effector CD8^+^ T cells by reducing avidity and polyfunctionality [43–45]. In relation to the role of STAT-6 in CTL responses, it has been reported that the activation and function of CTL may be impaired in STAT-6^−/−^ mice during infection with the intracellular parasite *Toxoplasma gondii*, likely as a consequence of reduced levels of CD86 expression on DC [46]. The demonstration that activation of CD8 T cells in STAT-6^−/−^ mice was restored after transfer of splenic adherent cells or bone-marrow derived DC from WT mice whereas transfer of naïve WT CD8 T cells into STAT-6^−/−^ mice were not activated suggested a defect in antigen presentation [46]. It is known that *in vitro*, IL-13, like IL-4, in combination with GM-CSF can induce generation of functional DC [47], suggesting that IL-13 may be important in differentiation of DC via STAT-6 signaling. We speculate that IL-4R/IL-13R-associated STAT-6 signaling might be important in CD8 T cell activation and function during ECTV infection, possibly through maturation of antigen presenting cells, such as dendritic cells (DC). Apart from promoting the expression of several cell surface molecules responsible for antigen presentation; including MHC II, CD80, and CD86, IL-4/STAT-6 signaling regulates T-cell proliferation by decreasing the expression of p27^Kip1^, a known cyclin-dependent kinase inhibitor. In CD8 cells, STAT6 is required for Tc2 differentiation as the production of IL-4 and IL-5 is completely lost with STAT6 deficiency (reviewed in [48]). Taken together, STAT6 is required for IL-4-stimulated T-cell functions.

That STAT-6 deficiency further exacerbates susceptibility of BALB/c mice to ECTV infection is in stark contrast to a previous report in which STAT-6^−/−^ mice were found to be more resistant than WT BALB/c mice due to augmented NK cell and IFN-γ responses [25]. In the current study, apart from a 2–3-fold increase in the cytolytic activity of NK cells in STAT-6^−/−^ mice, all other responses were either far worse or no different to WT mice. We believe that there may be a simple explanation for this discrepancy. In the present study we used STAT-6^−/−^ mice that were backcrossed to the BALB/c background for at least 10 generations [29]. In the previous study, STAT-6^−/−^ mice generated on a 129 background [49] and backcrossed to the BALB/c background for only 6 generations were used. Given that several of the 129 sub-strains are resistant to mousepox [10], we believe that the 129/Sv background genes may have influenced the outcome of infection in that study. It is likely that STAT-6 deficiency in ECTV-resistant C57BL/6 or 129 mouse strains might further augment Th1 responses but this is not the case in the ECTV-susceptible BALB/c mice. Consistent with this proposition, we have found that C57BL/6 mice deficient in IL-4 generate more robust CTL and IFN-γ responses which correlated with more efficient virus control compared to WT littermate controls (data not shown). Furthermore, when the STAT-6^−/−^ mice used in the previous study [25,49] were backcrossed to the BALB/c background 10–12 times, they were found to be more susceptible to ECTV-WT infection than WT littermates (data not shown). Clearly, STAT-6 deficiency does not increase the resistance of BALB/c mice to ECTV infection. On the contrary, STAT-6 deficiency further increased susceptibility of the BALB/c strain to ECTV.

Our finding that deficiencies in IL-4/IL-13/STAT-6 signaling pathways significantly reduced the numbers of IFN-γ producing CD4 and CD8 T cells indicates an absence of a switch to a Th1-like response and might be unique to this viral model. For example, Th1/Th2 cytokine production by IL-13^−/−^ cells is varied depending on *in vitro* or *in vivo* stimulation conditions and the type of cells involved [27]. IL-13 deficient splenocytes or CD4 T cells stimulated with mitogens produce reduced levels of IL-4 but similar levels of IFN-γ compared to WT cells. Under Th1/Th2 culture conditions, IL-13^−/−^ CD4 T cells produce dramatically reduced levels of IL-4 but only a 2-fold higher amount of IFN-γ. In contrast, mast cells from IL-13^−/−^ and WT mice stimulated with PMA and ionomycin produce comparable levels of IL-4. *In vivo*, challenge of IL-13^−/−^ mice with schistosome eggs reduced IL-4 levels only by about 3-fold whereas challenge with the parasite *N*. *brasiliensis* had no impact on Th2 response generation or IL-4 production by lymph node cells or purified CD4 T cells.

It is conceivable that regulation of Th2 response generation *in vivo* during infection with a virus is far more complex than how it is understood to occur in *in vitro* culture conditions. Various stimuli, cell types and signalling pathways are involved in orchestrating Th2 responses of which there are also variants [19,50–52]. Indeed, Th2 differentiation has been shown to occur in mice that are deficient in IL-4, IL-13, IL-4Rα or STAT-6 and cytokines like thymic stromal lymphopoietin, IL-25 and IL-33 can induce Th2 responses [19,27,51,52]. There is consensus, however, that GATA3 is required for this process, even if expressed at low levels [53–55]. In the current study, we found that numbers of GATA3^+^ CD4 T cells were highest in both WT and IL-4^−/−^ mice infected with WT virus but numbers were reduced in the GKO strains. These strains also had the highest number of T-bet^+^ CD4 T cells. T-bet can negatively regulate GATA3 [56] and in STAT-6^−/−^ mice, T-bet^+^ CD4 T cell numbers were increased with a corresponding decrease in GATA-3^+^ CD4 T cells. These changes, however, did not translate to an increase in IFN-γ^+^ CD4 T cells. Overall, there was little correlation between GATA-3 and IL-4 expression or T-bet and IFN-γ expression in CD4 T cells regardless of the type of infecting virus. We believe that the use of transgenic CD4 and CD8 T cells, specific for a model antigen, and deficient in IL-4, IL-13, IL-4Rα or STAT-6 might help better understand the molecular mechanisms involved in driving Th2 responses in BALB/c mice infected with ECTV. The adoptive transfer of such transgenic cells into recipient mice combined with the use of recombinant ECTV encoding the model antigen for infection is an approach that we propose to undertake.

## Conclusion

We have established that deficiency in specific factors associated with Th2 response development and/or the absence of vIFN-γ-bp during infection of BALB/c mice with ECTV does not bias development of a Th1 response. IL-4, STAT-6 and IL-13, which are normally associated with development of Th2 responses, play important and non-redundant roles in the generation of optimal antiviral CTL responses. Our data also established that the absence of IL-13 and STAT-6 further increased the susceptibility of BALB/c mice to ECTV infection. We conclude that host resistance or susceptibility to mousepox is far more complex and goes beyond the presence or absence of select genetic factors that control Th differentiation.

## Supporting Information

S1 Fig Representative flow cytometry profiles of IFN-γ^+^ 8 T cells from ECTV-WT infected mice. Data are from one of the three separate experiments showing intracellular IFN-γ expression (Y-axis) by splenic 8 T cells (X-axis) from WT and GKO mice. The numbers in the upper right quadrants in individual panels indicate percentages of IFN-γ-producing 8 T cells after stimulation with (from left) the irrelevant control (HSV-1 gB) peptide (first column), whole virus (ECTV) (second column), L^d^-EVM026 (third column), K^d^-EVMA52 (forth column) or D^d^-EVM043 peptides (fifth column). Absolute numbers of IFN-γ^+^ 8 T were obtained by multiplying the percentage of cells with the total number of splenocytes from each mouse for each strain.
(TIF)

S2 Fig Representative flow cytometry profiles of IFN-γ^+^ 8 T cells from ECTV-IFN-γbp^Δ^-infected mice. Data are from one of the three separate experiments showing intracellular IFN-γ expression (Y-axis) by splenic 8 T cells (X-axis) from WT and GKO mice. The numbers in the upper right quadrants in individual panels indicate percentages of IFN-γ-producing 8 T cells after stimulation with (from left) the irrelevant control (HSV-1 gB) peptide (first column), whole virus (ECTV) (second column), L^d^-EVM026 (third column), K^d^-EVMA52 (fourth column) or D^d^-EVM043 peptides (fifth column). Absolute numbers of IFN-γ^+^ 8 T were obtained by multiplying the percentage of cells with the total number of splenocytes from each mouse for each strain.
(TIF)

S3 Fig Representative flow cytometry profiles of IFN-γ^+^ CD4 T cells from uninfected (naïve) and virus-infected mice. Data are from one of three separate experiments showing intracellular IFN-γ expression (Y-axis) in unstimulated splenic CD4 T cells (X-axis) from naïve (first column), ECTV-WT- (second column) or ECTV-IFN-γbp^Δ^- (third column) infected WT and GKO mice. The numbers in the upper right quadrants in individual panels indicate percentages of IFN-γ-producing CD4 T cells.
(TIF)

S4 Fig Representative flow cytometry profiles of IL-4^+^ CD4 T cells from uninfected (naïve) and virus-infected mice. Data are from one of three separate experiments showing intracellular IL-4 expression (Y-axis) in unstimulated splenic CD4 T cells (X-axis) from naïve (first column), ECTV-WT- (second column) or ECTV-IFN-γbp^Δ^- (right column) infected WT and GKO mice. The numbers in the upper right quadrants in individual panels indicate percentages of IL-4-producing CD4 T cells.
(TIF)

S5 Fig Survival of ECTV-WT-infected GKO mice compared with wild type BALB/c mice. Data in this figure is the same as in Fig. 1 but presented to compare survival curves of each GKO strain with wild type BALB/c mice. *P* values were obtained by using Kaplan-Meier Log rank statistical test: *, *p* < 0.05.
(TIF)

S6 Fig Survival of ECTV-IFN-γbp^Δ^-infected GKO mice compared with wild type BALB/c mice. Data in this figure is the same as in Fig. 1 but presented to compare survival curves of each GKO strain with wild type BALB/c mice. *P* values were obtained by using Kaplan-Meier Log rank statistical test: *, *p* < 0.05.
(TIF)

S1 Table Ectromelia virus-specific CD8 T cell determinants. ^a^ EVM represents nomenclature for ECTV-specific 8 T cell determinants.
(DOCX)

S2 Table Statistical analysis for survival proportions at day 21 p.i. ^a^ ECTV-WT *vs*. ECTV-IFN-γbp^Δ^ *P value* using Logrank (Mantel-Cox) test. For extremely significant (****) P < 0.0001; extremely significant (***) 0.0001< P <0.001; very significant (**) 0.001< P <0.01; significant (*) 0.01< P <0.05; not significant (ns) P ≥ 0.05. ^b^ Number in brackets is the median survival time in days. ^c^ Number of animals in group. ^d^ BALB/c.WT *vs*. GKO strain *P value* using Logrank (Mantel-Cox) test. ^e^ undefined
(DOCX)

S3 Table Statistical analysis for viral load in livers of WT mice compared with GKO strains. ^a^ To evaluate significant differences between groups, viral titers were log transformed and 2-way ANOVA performed followed by Fisher’s LSD test. For extremely significant (****) P < 0.0001; extremely significant (***) 0.0001< P <0.001; very significant (**) 0.001< P <0.01; significant (*) 0.01< P <0.05; not significant (ns) P ≥ 0.05. ^b^ ECTV-WT *vs*. ECTV-IFN-γbp^Δ^. ^c^ BALB/c.WT *vs*. GKO strain.
(DOCX)

S4 Table Statistical analysis for viral load in spleens of WT mice compared with GKO strains. ^a^ To evaluate significant differences between groups, viral titers were log transformed and 2-way ANOVA performed followed by Fisher’s LSD test. For extremely significant (****) P < 0.0001; extremely significant (***) 0.0001< P <0.001; very significant (**) 0.001< P <0.01; significant (*) 0.01< P <0.05; not significant (ns) P ≥ 0.05. ^b^ ECTV-WT *vs*. ECTV-IFN-γbp^Δ^. ^c^ BALB/c.WT *vs*. GKO.
(DOCX)

S5 Table Statistical analysis for viral load in blood of WT mice compared with GKO strains. ^a^ To evaluate significant differences between groups, viral titres were log transformed and 2-way ANOVA performed followed by Fisher’s LSD test. For extremely significant (****) P < 0.0001; extremely significant (***) 0.0001< P <0.001; very significant (**) 0.001< P <0.01; significant (*) 0.01< P <0.05; not significant (ns) P ≥ 0.05. ^b^ ECTV-WT *vs*. ECTV-IFN-γbp^Δ^. ^c^ BALB/c.WT *vs*. GKO strain.
(DOCX)
