# Supplementary figures and images for: Deficiency in Th2 Cytokine Responses Exacerbate Orthopoxvirus Infection
Source: PLoS One. 2015 Mar 9;10(3):e0118685. doi: 10.1371/journal.pone.0118685 (PMC4353717; doi:10.1371/journal.pone.0118685)

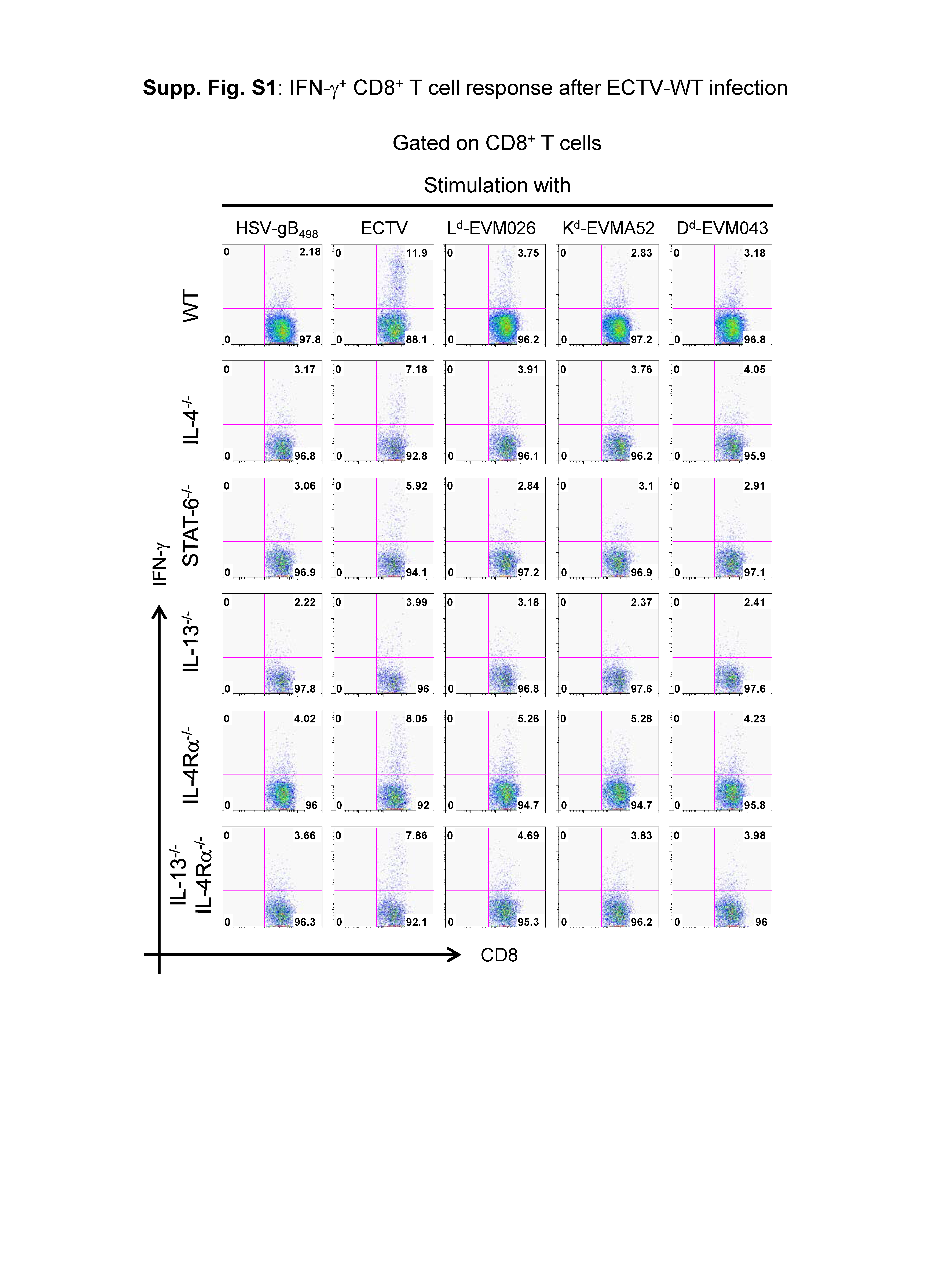

Supplement: S1 Fig — Data are from one of the three separate experiments showing intracellular IFN-γ expression (Y-axis) by splenic 8 T cells (X-axis) from WT and GKO mice. The numbers in the upper right quadrants in individual panels indicate percentages of IFN-γ-producing 8 T cells after stimulation with (from left) the irrelevant control (HSV-1 gB) peptide (first column), whole virus (ECTV) (second column), Ld-EVM026 (third column), Kd-EVMA52 (forth column) or Dd-EVM043 peptides (fifth column). Absolute numbers of IFN-γ+ 8 T were obtained by multiplying the percentage of cells with the total number of splenocytes from each mouse for each strain. (TIF) [file pone.0118685.s001.tif]

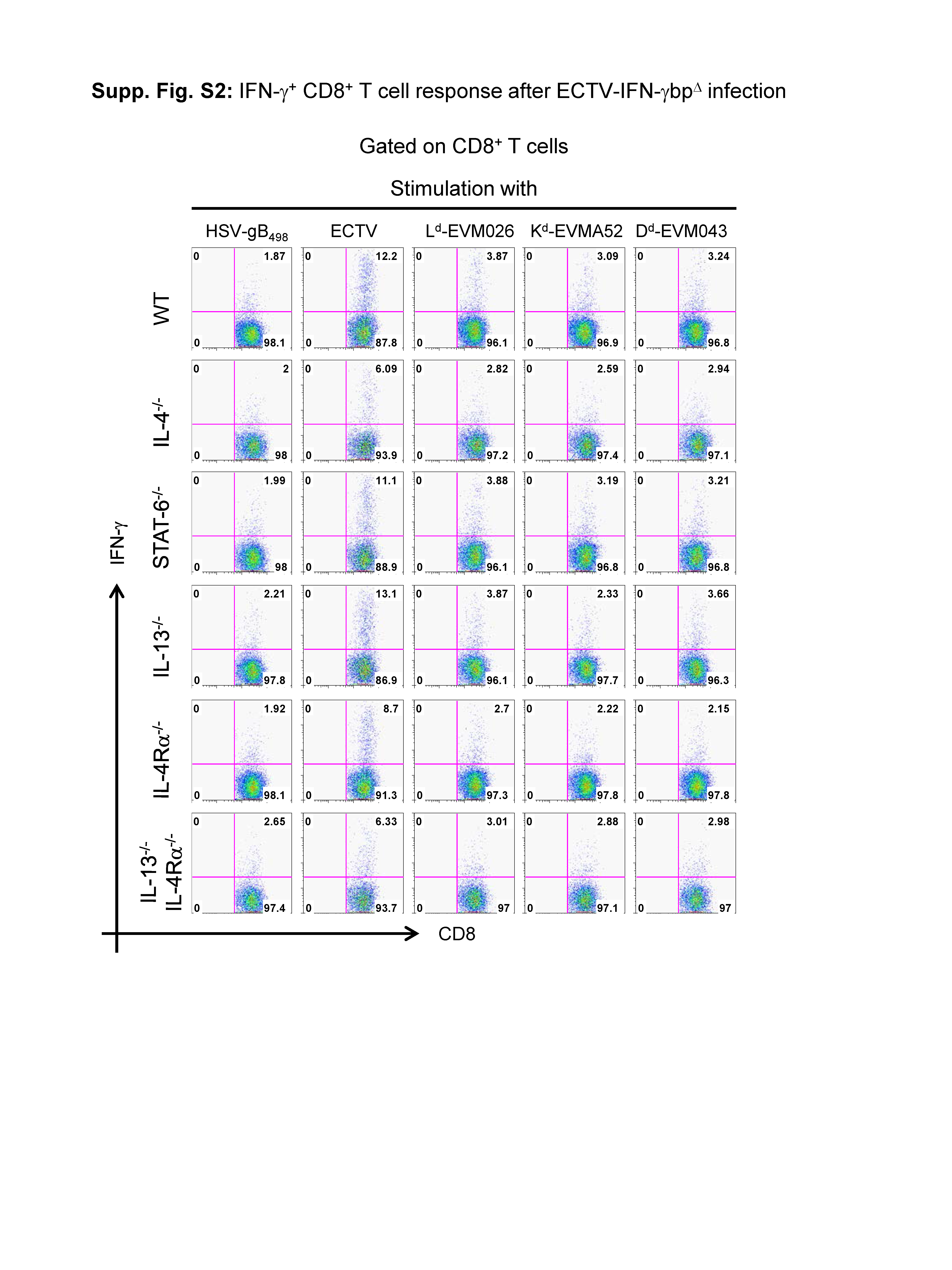

Supplement: S2 Fig — Data are from one of the three separate experiments showing intracellular IFN-γ expression (Y-axis) by splenic 8 T cells (X-axis) from WT and GKO mice. The numbers in the upper right quadrants in individual panels indicate percentages of IFN-γ-producing 8 T cells after stimulation with (from left) the irrelevant control (HSV-1 gB) peptide (first column), whole virus (ECTV) (second column), Ld-EVM026 (third column), Kd-EVMA52 (fourth column) or Dd-EVM043 peptides (fifth column). Absolute numbers of IFN-γ+ 8 T were obtained by multiplying the percentage of cells with the total number of splenocytes from each mouse for each strain. (TIF) [file pone.0118685.s002.tif]

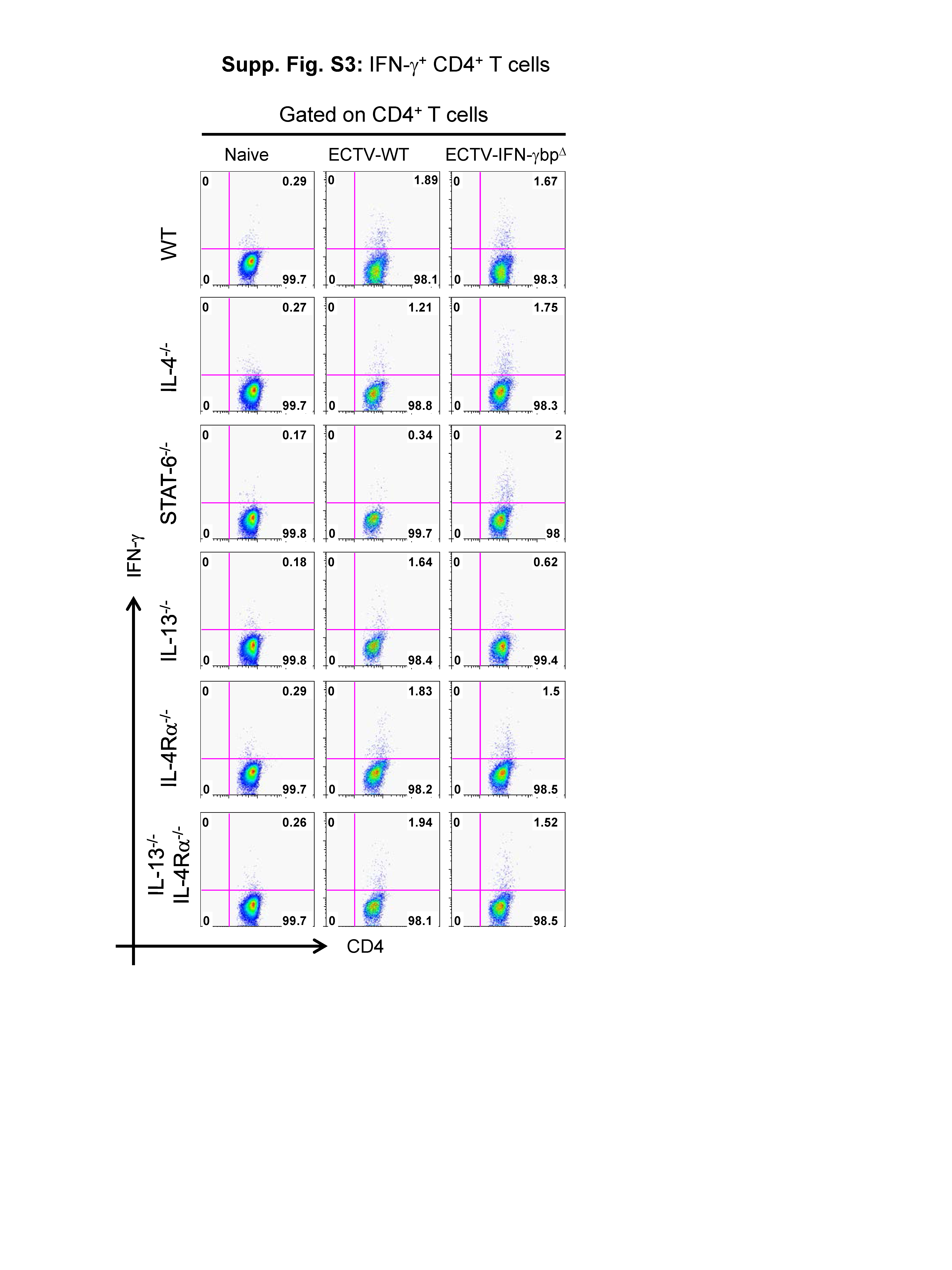

Supplement: S3 Fig — Data are from one of three separate experiments showing intracellular IFN-γ expression (Y-axis) in unstimulated splenic CD4 T cells (X-axis) from naïve (first column), ECTV-WT- (second column) or ECTV-IFN-γbpΔ- (third column) infected WT and GKO mice. The numbers in the upper right quadrants in individual panels indicate percentages of IFN-γ-producing CD4 T cells. (TIF) [file pone.0118685.s003.tif]

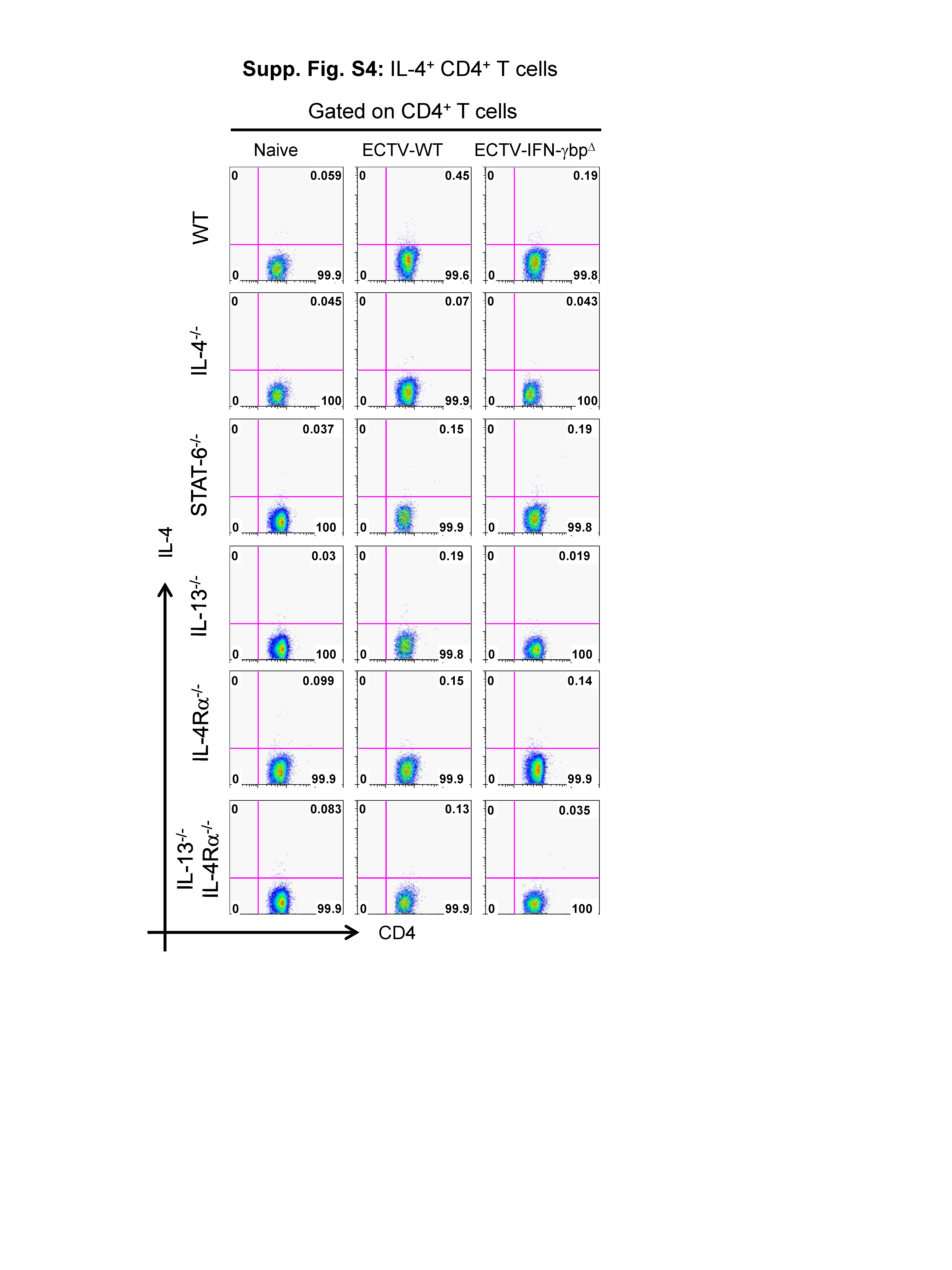

Supplement: S4 Fig — Data are from one of three separate experiments showing intracellular IL-4 expression (Y-axis) in unstimulated splenic CD4 T cells (X-axis) from naïve (first column), ECTV-WT- (second column) or ECTV-IFN-γbpΔ- (right column) infected WT and GKO mice. The numbers in the upper right quadrants in individual panels indicate percentages of IL-4-producing CD4 T cells. (TIF) [file pone.0118685.s004.tif]

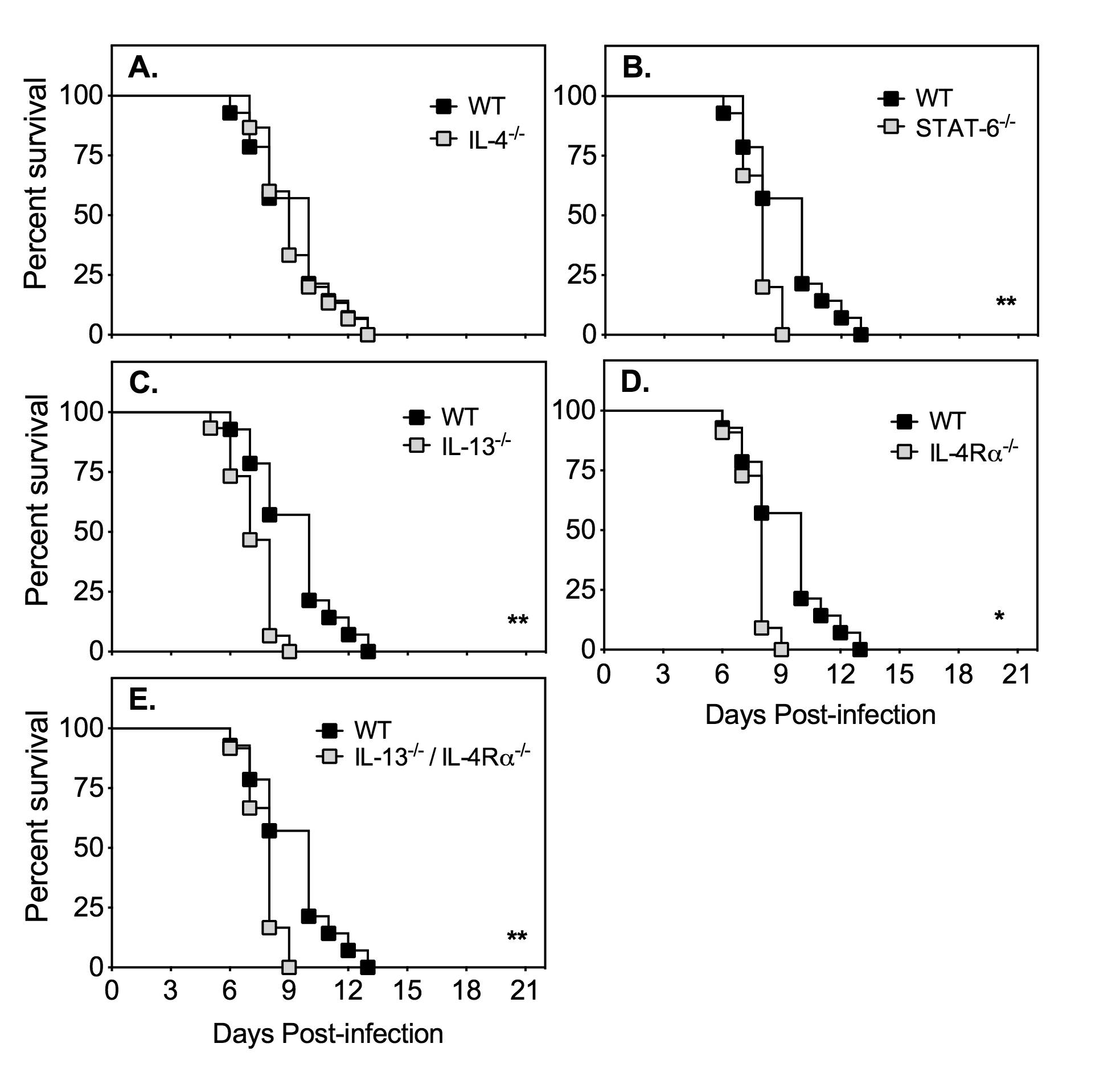

Supplement: S5 Fig — Data in this figure is the same as in Fig. 1 but presented to compare survival curves of each GKO strain with wild type BALB/c mice. P values were obtained by using Kaplan-Meier Log rank statistical test: *, p < 0.05. (TIF) [file pone.0118685.s005.tif]

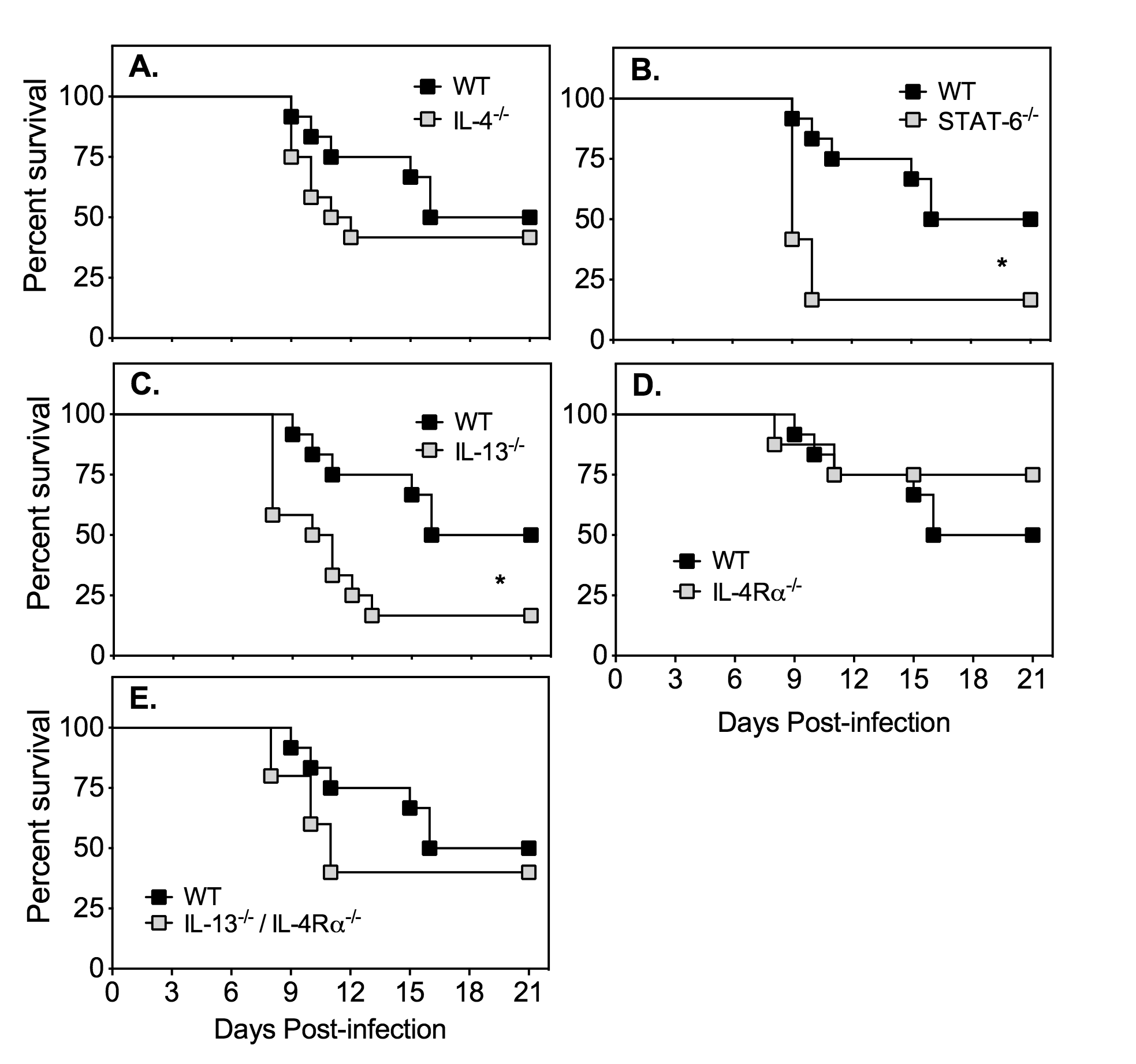

Supplement: S6 Fig — Data in this figure is the same as in Fig. 1 but presented to compare survival curves of each GKO strain with wild type BALB/c mice. P values were obtained by using Kaplan-Meier Log rank statistical test: *, p < 0.05. (TIF) [file pone.0118685.s006.tif]
