# Supplementary material for: Deficiency in Th2 Cytokine Responses Exacerbate Orthopoxvirus Infection
Source: PLoS One. 2015 Mar 9;10(3):e0118685. doi: 10.1371/journal.pone.0118685 (PMC4353717; doi:10.1371/journal.pone.0118685)
Supplement: S1 Table — a EVM represents nomenclature for ECTV-specific 8 T cell determinants. (DOCX) [file pone.0118685.s007.docx]

**Table S1. Ectromelia virus-specific CD8 T cell determinants**

| **Gene** | **Determinant name** ^a^ | **Peptide sequence** | **Restricted by**  **MHC class I** |
| --- | --- | --- | --- |
| ECTV-MOS-026 | EVM026_26-34_ | SNHAAGYD | L^d^ |
| ECTV-MOS-043 | EVM043_140-148_ | VGPSNSPIF | K^d^ |
| ECTV-MOS-A52R | EVMA52_65-73_ | KYGRLFNEI | D^d^ |

^a^ EVM represents nomenclature for ECTV-specific CD8^+^ T cell determinants.
