# Supplementary material for: Deficiency in Th2 Cytokine Responses Exacerbate Orthopoxvirus Infection
Source: PLoS One. 2015 Mar 9;10(3):e0118685. doi: 10.1371/journal.pone.0118685 (PMC4353717; doi:10.1371/journal.pone.0118685)
Supplement: S2 Table — a ECTV-WT vs. ECTV-IFN-γbpΔ P value using Logrank (Mantel-Cox) test. For extremely significant (****) P < 0.0001; extremely significant (***) 0.0001< P <0.001; very significant (**) 0.001< P <0.01; significant (*) 0.01< P <0.05; not significant (ns) P ≥ 0.05. b Number in brackets is the median survival time in days. c Number of animals in group. d BALB/c.WT vs. GKO strain P value using Logrank (Mantel-Cox) test. e undefined (DOCX) [file pone.0118685.s008.docx]

**Table S2: Statistical analysis for survival proportions at day 21 p.i.**

| **Virus** | **ECTV-WT** | | | **ECTV-IFN-γbp^Δ^** | | | ***Significance , P value ^a^*** |
| --- | --- | --- | --- | --- | --- | --- | --- |
| **WT** | 0.0% | (10.0)^b^ | n^c^=14 | 50.0% | (18.5) | n=12 | *****, <0.0001* |
| **IL-4^-/-^** | 0.0% | (9.0) | n=15 | 41.7% | (11.5) | n=12 | ***, 0.0025* |
| ***P value ^d^*** | ns, *0.7359* | | | ns, *0.4445* | | |  |
| **STAT-6^-/-^** | 0.0% | (8.0) | n=15 | 16.7% | (9.0) | n=12 | *****, <0.0001* |
| ***P value ^d^*** | ***, 0.0055* | | | **, 0.0140* | | |  |
| **IL-13^-/-^** | 0.0% | (7.0) | n=15 | 16.7% | (10.5) | n=12 | ****, 0.0001* |
| ***P value ^d^*** | ***, 0.0011* | | | **, 0.0213* | | |  |
| **IL-4Rα^-/-^** | 0.0% | (8.0) | n=11 | 75.0% | (un)^e^ | n=8 | ****, 0.0002* |
| ***P value ^d^*** | **, 0.0125* | | | ns, *0.3963* | | |  |
| **IL-13^-/-^/IL-4Rα^-/-^** | 0.0% | (8.0) | n=8 | 40.0% | (11.0) | n=5 | ***, 0.0023* |
| ***P value ^d^*** | ***, 0.0080* | | | ns, *0.4753* | | |  |

^a^ ECTV-WT *vs.* ECTV-IFN-γbp^Δ^ *P value* using Logrank (Mantel-Cox) test. For extremely significant (****) P < 0.0001; extremely significant (***) 0.0001< P <0.001; very significant (**) 0.001< P <0.01; significant (*) 0.01< P <0.05; not significant (ns) P ≥ 0.05.

^b^ Number in brackets is the median survival time in days

^c^ Number of animals in group

^d^ BALB/c.WT *vs.* GKO strain *P value* using Logrank (Mantel-Cox) test

^e^ undefined
