# Supplementary material for: Deficiency in Th2 Cytokine Responses Exacerbate Orthopoxvirus Infection
Source: PLoS One. 2015 Mar 9;10(3):e0118685. doi: 10.1371/journal.pone.0118685 (PMC4353717; doi:10.1371/journal.pone.0118685)
Supplement: S3 Table — a To evaluate significant differences between groups, viral titers were log transformed and 2-way ANOVA performed followed by Fisher’s LSD test. For extremely significant (****) P < 0.0001; extremely significant (***) 0.0001< P <0.001; very significant (**) 0.001< P <0.01; significant (*) 0.01< P <0.05; not significant (ns) P ≥ 0.05. b ECTV-WT vs. ECTV-IFN-γbpΔ. c BALB/c.WT vs. GKO strain. (DOCX) [file pone.0118685.s009.docx]

**Table S3: Statistical analysis for viral load in livers of WT mice compared with GKO strains**

| **Virus** | **ECTV-WT** | **ECTV-IFN-γbp^Δ^** | ***Significance ^a^, P value ^b^*** |
| --- | --- | --- | --- |
|  | **Log_10_ virus titer** (Mean ± SD)/g tissue | |  |
| **WT** | 5.686 ± 0.707 | 5.423 ± 0.407 | ns, 0.4853 |
| **IL-4^-/-^** | 5.125 ± 0.811 | 4.583 ± 0.696 | ns, 0.1545 |
| ***Significance, P value ^c^*** | ns, 0.1408 | *, 0.0298 |  |
| **STAT-6^-/-^** | 6.617 ± 0.629 | 4.830 ± 0.551 | ****, < 0.0001 |
| ***Significance, P value ^c^*** | *, 0.0168 | ns, 0.1425 |  |
| **IL-13^-/-^** | 6.289 ± 0.264 | 4.943 ± 1.006 | ***, 0.0008 |
| ***Significance, P value ^c^*** | ns, 0.1148 | ns, 0.2072 |  |
| **IL-4Rα^-/-^** | 6.033 ± 0.417 | 4.500 ± 0.515 | ***, 0.0002 |
| ***Significance, P value ^c^*** | ns, 0.3599 | *, 0.0272 |  |
| **IL-13/IL-4Rα^-/-^** | 4.933 ± 0.217 | 4.098 ± 0.361 | *, 0.0309 |
| ***Significance, P value ^c^*** | ns, 0.0501 | ***, 0.0009 |  |

^a^ To evaluate significant differences between groups, viral titers were log transformed and 2-way ANOVA performed followed by Fisher’s LSD test. For extremely significant (****) P < 0.0001; extremely significant (***) 0.0001< P <0.001; very significant (**) 0.001< P <0.01; significant (*) 0.01< P <0.05; not significant (ns) P ≥ 0.05.

^b^ ECTV-WT *vs.* ECTV-IFN-γbp^Δ^

^c^ BALB/c.WT *vs.* GKO strain
