# Supplementary material for: Deficiency in Th2 Cytokine Responses Exacerbate Orthopoxvirus Infection
Source: PLoS One. 2015 Mar 9;10(3):e0118685. doi: 10.1371/journal.pone.0118685 (PMC4353717; doi:10.1371/journal.pone.0118685)
Supplement: S4 Table — a To evaluate significant differences between groups, viral titers were log transformed and 2-way ANOVA performed followed by Fisher’s LSD test. For extremely significant (****) P < 0.0001; extremely significant (***) 0.0001< P <0.001; very significant (**) 0.001< P <0.01; significant (*) 0.01< P <0.05; not significant (ns) P ≥ 0.05. b ECTV-WT vs. ECTV-IFN-γbpΔ. c BALB/c.WT vs. GKO. (DOCX) [file pone.0118685.s010.docx]

**Table S4: Statistical analysis for viral load in spleens of WT mice compared with GKO strains**

| **Virus** | **ECTV-WT** | **ECTV-IFN-γbp^Δ^** | ***Significance ^a^, P value ^b^*** |
| --- | --- | --- | --- |
|  | **Log_10_ virus titer** (Mean ± SD)/g tissue | |  |
| **WT** | 7.292 ± 0.413 | 7.280 ± 0.223 | ns, 0.9511 |
| **IL-4^-/-^** | 7.692 ± 0.382 | 7.748 ± 0.208 | ns, 0.7884 |
| ***Significance, P value ^c^*** | ns, 0.0574 | *, 0.0272 |  |
| **STAT-6^-/-^** | 8.201 ± 0.291 | 7.339 ± 0.346 | ***, 0.0003 |
| ***Significance, P value ^c^*** | ****, < 0.0001 | ns, 0.7847 |  |
| **IL-13^-/-^** | 7.886 ± 0.117 | 7.457 ± .508 | *, 0.0422 |
| ***Significance, P value ^c^*** | **, 0.0058 | ns, 0.3927 |  |
| **IL-4Rα^-/-^** | 8.057 ± 0.380 | 7.862 ± 0.363 | ns, 0.3451 |
| ***Significance, P value ^c^*** | ***, 0.0005 | **, 0.0068 |  |
| **IL-13^-/-^/IL-4Rα^-/-^** | 7.386 ± 0.380 | 7.322 ± 0.303 | ns, 0.7564 |
| ***Significance, P value ^c^*** | ns, 0.6502 | ns, 0.8376 |  |

^a^ To evaluate significant differences between groups, viral titers were log transformed and 2-way ANOVA performed followed by Fisher’s LSD test. For extremely significant (****) P < 0.0001; extremely significant (***) 0.0001< P <0.001; very significant (**) 0.001< P <0.01; significant (*) 0.01< P <0.05; not significant (ns) P ≥ 0.05.

^b^ ECTV-WT *vs.* ECTV-IFN-γbp^Δ^

^c^ BALB/c.WT *vs.* GKO
