# Supplementary material for: Deficiency in Th2 Cytokine Responses Exacerbate Orthopoxvirus Infection
Source: PLoS One. 2015 Mar 9;10(3):e0118685. doi: 10.1371/journal.pone.0118685 (PMC4353717; doi:10.1371/journal.pone.0118685)
Supplement: S5 Table — a To evaluate significant differences between groups, viral titres were log transformed and 2-way ANOVA performed followed by Fisher’s LSD test. For extremely significant (****) P < 0.0001; extremely significant (***) 0.0001< P <0.001; very significant (**) 0.001< P <0.01; significant (*) 0.01< P <0.05; not significant (ns) P ≥ 0.05. b ECTV-WT vs. ECTV-IFN-γbpΔ. c BALB/c.WT vs. GKO strain. (DOCX) [file pone.0118685.s011.docx]

**Table S5: Statistical analysis for viral load in blood of WT mice compared with GKO strains**

| **Virus** | **ECTV-WT** | **ECTV-IFN-γbp^Δ^** | ***Significance ^a^, P value ^b^*** |
| --- | --- | --- | --- |
|  | **Log_10_ virus titer** (Mean ± SD)/g blood | |  |
| **WT** | 3.102 ± 0.659 | 2.583 ± 0.601 | ns, 0.1510 |
| **IL-4^-/-^** | 3.703 ± 0.321 | 2.903 ± 0.335 | *, 0.0290 |
| ***Significance, P value ^c^*** | ns, 0.1971 | ns, 0.3728 |  |
| **STAT-6^-/-^** | 4.537 ± 0.919 | 3.028 ± 0.333 | ***, 0.0002 |
| ***Significance, P value ^c^*** | ***, 0.0002 | ns, 0.2435 |  |
| **BALB/c.IL-13^-/-^** | 3.565 ± 0.310 | 3.579 ± 0.231 | ns, 0.9702 |
| ***Significance, P value ^c^*** | ns, 0.1978 | **, 0.0073 |  |
| **IL-4Rα^-/-^** | 3.258 ± 0.776 | 3.084 ± 0.844 | ns, 0.6252 |
| ***Significance, P value ^c^*** | ns, 0.6610 | ns, 0.1653 |  |
| **IL-13/IL-4Rα^-/-^** | 2.473 ± 0.535 | 2.739 ± 0.117 | ns, 0.4575 |
| ***Significance, P value ^c^*** | ns, 0.0830 | ns, 0.6634 |  |

^a^ To evaluate significant differences between groups, viral titres were log transformed and 2-way ANOVA performed followed by Fisher’s LSD test. For extremely significant (****) P < 0.0001; extremely significant (***) 0.0001< P <0.001; very significant (**) 0.001< P <0.01; significant (*) 0.01< P <0.05; not significant (ns) P ≥ 0.05.

^b^ ECTV-WT *vs.* ECTV-IFN-γbp^Δ^

^c^ BALB/c.WT *vs.* GKO strain
